# Effectiveness and safety of EVT in patients with acute LVO and low NIHSS

**DOI:** 10.3389/fneur.2022.955725

**Published:** 2022-08-05

**Authors:** Beom Joon Kim, Bijoy K. Menon, Joonsang Yoo, Jung Hoon Han, Bum Joon Kim, Chi Kyung Kim, Jae Guk Kim, Joon-Tae Kim, Hyungjong Park, Sung Hyun Baik, Moon-Ku Han, Jihoon Kang, Jun Yup Kim, Keon-Joo Lee, Jong-Moo Park, Kyusik Kang, Soo Joo Lee, Jae-Kwan Cha, Dae-Hyun Kim, Jin-Heon Jeong, Tai Hwan Park, Sang-Soon Park, Kyung Bok Lee, Jun Lee, Keun-Sik Hong, Yong-Jin Cho, Hong-Kyun Park, Byung-Chul Lee, Kyung-Ho Yu, Mi-Sun Oh, Dong-Eog Kim, Wi-Sun Ryu, Kang-Ho Choi, Jay Chol Choi, Joong-Goo Kim, Jee-Hyun Kwon, Wook-Joo Kim, Dong-Ick Shin, Kyu Sun Yum, Sung-Il Sohn, Jeong-Ho Hong, Chulho Kim, Sang-Hwa Lee, Juneyoung Lee, Mohammed A. Almekhlafi, Andrew Demchuk, Hee-Joon Bae

**Affiliations:** ^1^Department of Neurology and Cerebrovascular Center, Seoul National University College of Medicine, Seoul National University Bundang Hospital, Seongnam-si, South Korea; ^2^Calgary Stroke Program, Department of Clinical Neuroscience, Radiology and Community Health Sciences, University of Calgary, Calgary, AB, Canada; ^3^Department of Neurology, Yongin Severance Hospital, Yongin-si, South Korea; ^4^Department of Neurology, Korea University Guro Hospital, Seoul, South Korea; ^5^Department of Neurology, Asan Medical Center, Seoul, South Korea; ^6^Department of Neurology, Eulji University Hospital, Daejeon, South Korea; ^7^Department of Neurology, Chonnam National University Hospital, Gwangju, South Korea; ^8^Department of Neurology, Keimyung University Dongsan Medical Center, Daegu, South Korea; ^9^Department of Radiology, Seoul National University Bundang Hospital, Seongnam-si, South Korea; ^10^Department of Neurology, Uijeongbu Eulji Medical Center, Eulji University School of Medicine, Uijeongbu-si, South Korea; ^11^Department of Neurology, Nowon Eulji Medical Center, Eulji University School of Medicine, Seoul, South Korea; ^12^Department of Neurology, Dong-A University Hospital, Busan, South Korea; ^13^Department of Neurology, Seoul Medical Center, Seoul, South Korea; ^14^Department of Neurology, Soonchunhyang University Hospital, Seoul, South Korea; ^15^Department of Neurology, Yeungnam University Medical Center, Daegu, South Korea; ^16^Department of Neurology, Inje University Ilsan Paik Hospital, Goyang-si, South Korea; ^17^Department of Neurology, Hallym University Sacred Heart Hospital, Anyang-si, South Korea; ^18^Department of Neurology, Dongguk University Ilsan Hospital, Goyang-si, South Korea; ^19^Department of Neurology, Jeju National University Hospital, Jeju, South Korea; ^20^Department of Neurology, Ulsan University Hospital, Ulsan, South Korea; ^21^Department of Neurology, Chungbuk National University Hospital, Cheongju-si, South Korea; ^22^Department of Neurology, Hallym University Chuncheon Sacred Heart Hospital, Chuncheon-si, South Korea; ^23^Department of Biostatistics, Korea University, Seoul, South Korea

**Keywords:** endovascular recanalization, mild stroke, low NIHSS score, early neurological deterioration, CRCS-K, multicenter registry

## Abstract

**Background and purpose:**

There is much uncertainty in endovascular treatment (EVT) decisions in patients with acute large vessel occlusion (LVO) and mild neurological deficits.

**Methods:**

From a prospective, nationwide stroke registry, all patients with LVO and baseline NIHSS <6 presenting within 24 h from the time last known well (LKW) were included. Early neurological deterioration (END) developed before EVT was prospectively collected as an increasing total NIHSS score ≥2 or any worsening of the NIHSS consciousness or motor subscores during hospitalization not related to EVT. Significant hemorrhage was defined as PH2 hemorrhagic transformation or hemorrhage at a remote site. The modified Rankin Scale (mRS) was prospectively collected at 3 months.

**Results:**

Among 1,083 patients, 149 (14%) patients received EVT after a median of 5.9 [3.6–12.3] h after LKW. In propensity score-matched analyses, EVT was not associated with mRS 0-1 (matched OR 0.99 [0.63–1.54]) but increased the risk of a significant hemorrhage (matched OR, 4.51 [1.59–12.80]). Extraneous END occurred in 207 (19%) patients after a median of 24.5 h [IQR, 13.5–41.9 h] after LKW (incidence rate, 1.41 [95% CI, 1.23–1.62] per 100 person-hours). END unrelated to EVT showed a tendency to modify the effectiveness of EVT (P-for-interaction, 0.08), which decreased the odds of having mRS 0–1 in mild LVO patients without END (adjusted OR, 0.63 [0.40–0.99]).

**Conclusions:**

The use of EVT in patients with acute LVO and low NIHSS scores may require the assessment of individual risks of early deterioration, hemorrhagic complications and expected benefit.

## Introduction

Multiple recent randomized clinical trials (RCTs) have demonstrated the superiority of endovascular treatment (EVT) over medical management in ischemic stroke patients with anterior circulation large vessel occlusions (LVOs) presenting within 24 h from the time last known well (LKW) (1). The majority of these RCTs excluded patients with mild neurological deficits who comprise a substantial proportion of acute ischemic stroke patients due to LVO (2). Despite their non-disabling presentation, the clinical outcomes of mild LVO patients are not as favorable as expected (3). Several recent observational studies have reported that EVT may be associated with increased hemorrhagic complications and no overall clinical benefit in these patients (4–6). However, other observational studies have suggested potential benefits of EVT in these patients (7–11). More robust evidence from large high-quality registries is needed to help physicians make treatment decisions in these patients until ongoing trials present their results in a few years.

There is much uncertainty in the treatment decision for mild LVO patients. The decision to offer EVT is based on a consideration of the risks and benefits, and the latter of which is likely to be limited due to the mild neurological deficit at presentation. However, the early clinical course of mild LVO patients is dynamic, and early neurological deterioration (END) occurs in 10 to 20% of patients (12–15). Because END occurs some time after admission, these patients are often ineligible to receive recanalization treatments, and in-hospital logistics for emergent treatment tend to be delayed (16). These patients may also have received thrombolytics or antithrombotics that increase the risk of treatment-related hemorrhage. However, the ability to predict who among these patients is at higher risk of END is still limited.

Using prospectively collected data from the South Korean national registry of consecutive ischemic stroke patients [Clinical Research Collaboration for Stroke in Korea (CRCS-K)], we analyzed detailed clinical and imaging information in patients presenting with acute LVO but with mild stroke symptoms. We evaluated the effectiveness of EVT for mild LVO patients and then examined whether the effectiveness differs due to END that occurs before endovascular treatment.

## Methods

### Study design and population

Data were obtained from the CRCS-K registry, an ongoing, nationwide, multicenter acute stroke registry that prospectively sources records of patients with acute stroke or transient ischemic attack (TIA) admitted within seven days of onset (17). Between January 1, 2015 and March 31, 2019, a total of 36,339 admissions were recorded in the CRCS-K registry database. Among these admissions, patients who (1) reached hospital within 24 h after the time last known well (*n* = 24,596), (2) had a baseline NIHSS score <6 points (*n* = 15,436) and (3) had anterior circulation LVO (ICA or M1 or proximal M2 segment of MCA) confirmed by neuroimaging (*n* = 1083) were included (Supplementary Material I). Patients were treated per institutional protocols based on national guidelines at the time of practice (18, 19). The local institutional review boards (IRBs) of all participating centers of the CRCS-K registry approved the study with a waiver of consent. Secondary use of the registry data and additional review of medical records for this study were approved by IRBs [B-2007-622-105].

### Clinical data and outcome collection

The baseline demographics of all included subjects were retrieved from the CRCS-K registry database. The data definitions have been published elsewhere (17). Stroke etiology was classified by the Trial of Org 10172 in Acute Stroke Treatment with an MRI-based algorithm (20, 21). END was defined as any new neurological symptoms or signs that satisfied one or more of the following conditions: an increase in the total NIHSS score ≥2; an increase in the NIHSS 1a, 1b, or 1c subscore (level of consciousness) ≥1; or an increase in the NIHSS 5a, 5b, 6a or 6b subscore (motor) ≥1. END was also defined as any new neurological symptoms or signs that occurred during hospitalization directly due to the index stroke (17, 22). Only ENDs that occurred before the initiation of EVT or in medically treated patients were analyzed for the present study. END was further categorized by the NIHSS score increase at the time of END as mild END (NIHSS increase <4) and severe END (NIHSS score increase ≥4). Data on END were prospectively collected. The modified Rankin Scale (mRS), recurrent stroke and death up to 3 months after stroke were prospectively collected during a regular clinic visit or through a structured telephone interview conducted by an appropriately trained nurse.

### Central imaging core lab and image analysis

All neuroimaging data were retrospectively collected and independently evaluated by a central imaging core lab. Images were assessed for the Alberta Stroke Program Emergent CT Score (ASPECTS), collateral grade, location of cerebral arterial occlusion, tandem occlusion, white matter hyperintensities (Fazekas grade), old infarction, cerebral microbleeds, angiographic collateral grading (ASITN/SIR) and expanded treatment in cerebral ischemia after endovascular treatment. The collateral grade was categorized as good (grade 4 or 5), intermediate (2 or 3) or poor (0 or 1). Advanced white matter hyperintensity was defined as patients having Fazekas grade 2 or 3. Hemorrhages on follow-up CT or MR images were evaluated by the Heidelberg bleeding classification (HBC) (23); parenchymal hemorrhage 2 and/or HBC class III hemorrhages were deemed significant.

The central image lab consisted of vascular neurologists (JHH, BJK, BJK, CKK and J-TK), interventional neurologists (JGK, HP and JSY) and interventional radiologists (SHB). All images were independently evaluated by at least two raters (Supplementary Material II). Any discrepancy in reading between raters was adjudicated by a panel (BJK, JSY and SHB) to reach the final assessment. Details on image collection, anonymization, storage and back-up as well as image reading processes are provided in Supplementary Material III.

### Statistical analyses

Statistical analyses were performed based on a prespecified plan (Supplementary Material IV). Baseline patient characteristics were summarized and compared using chi-squared tests for categorical data, independent *t* tests for parametric data and a test of medians for non-parametric data. To estimate the association between baseline variables and END occurrence, a multivariable logistic regression model was constructed using clinically relevant variables or with univariate *P* values < 0.20. To compare patients who received EVT vs. those who did not, a propensity score (PS) for receiving EVT was estimated from clinically relevant variables (Supplementary Material V). The primary clinical outcome for analysis was 90-day mRS 0-1. Patients who received EVT and controls who received medical management were matched 1:1 with a caliper of 0.2 and without replacement. The inverse probability of receiving EVT was estimated and used as an individual weight in a separate model with robust sandwich estimators for standard errors (24). Covariates with standardized mean differences of 0.20 or more after weighting were further incorporated into this model (25). Crude and multivariable logistic regression models without the PS techniques were built as sensitivity analyses. The effectiveness and safety of EVT were also tested against various outcome indices. Pre-specified subgroup analyses were performed using multivariable logistic regression models for mRS 0–1 without propensity score balancing. Significance levels were set at *P* < 0.05 for two-tailed tests, except when testing for multiplicative interactions when a two-tailed *P* < 0.10 was considered statistically significant (26). All tests were considered exploratory and hypothesis-generating; no adjustment was therefore made for multiple tests (27). All statistical analyses were performed using R, version 4.0.2 (R Foundation for Statistical Computing).

## Results

### Baseline characteristics

Among 36,339 admissions to 16 hospitals over 4 years, 1,083 patients met the eligibility criteria with an mean age of 67.2 years ± 13.1 years, 669 males (62%), a median NIHSS score of 2 points [interquartile range (IQR), 1–4] and a median LKW to hospital arrival time of 4.3 h [1.6–10.9]. Among these patients, 223 (21%) had extracranial internal carotid artery (ICA) occlusion, 69 (6%) had intracranial ICA occlusion, 377 (35%) had proximal middle cerebral artery (M1) occlusion and 337 (31%) had distal middle cerebral artery (M2 and beyond) occlusions. Tandem occlusions (combined extracranial ICA and M1 or M2 occlusions) were found in 77 (7%) patients. A good collateral grade was found in 154 (75%) patients with extracranial ICA occlusion, 34 (51%) patients with intracranial ICA occlusion, 134 (38%) patients with M1 segment MCA occlusion, 218 (67%) patients with proximal M2 segment MCA occlusion and 33 (46%) patients with tandem occlusions. Excellent functional status (90-day mRS 0–1) was observed in 576 (54%) patients, while mortality within 90 days was noted in 36 (3%) patients (Table 1 and Supplementary Figure I).

**Table 1:** Clinical profiles of patients stratified according to patients receiving or not receiving endovascular treatment.

**Variables**	**All patients (n, 1083)**	**EVT (-) 934 (86.2%)**	**EVT (+) 149 (13.8%)**	**P-for-difference**
Demographics				
Age (yrs, SD)	67.2 ± 13.1	67.6 ± 13.2	64.8 ± 12.6	0.02
Male sex (*n*, %)	669 (61.8%)	574 (61.5%)	95 (63.8%)	0.66
Prestroke dependency (*n*, %)	186 (18.1%)	161 (18.1%)	25 (18.4%)	0.99
Stroke information				
Stroke mechanism (*n*, %)				<0.01
Large artery atherosclerosis	386 (38.2%)	344 (36.8%)	42 (28.2%)	
Cardioembolism	328 (32.5%)	275 (29.4%)	53 (35.6%)	
Other determined etiology	47 (4.7%)	41 (4.4%)	6 (4.0%)	
Undetermined etiology	249 (24.7%)	201 (21.5%)	48 (32.2%)	
TIA as an index stroke (*n*, %)	73 (6.7%)	73 (6.7%)	0	
Occluded artery				<0.01
Extracranial ICA	223 (20.6%)	203 (21.7%)	20 (13.4%)	
Intracranial ICA	69 (6.4%)	51 (5.5%)	18 (12.1%)	
M1	377 (34.8%)	308 (33.0%)	67 (46.3%)	
M2 or distal	337 (31.1%)	301 (32.2%)	36 (24.2%)	
Tandem occlusion	77 (7.1%)	71 (7.6%)	6 (4.0%)	
Baseline NIHSS score	2 [1–4]	2 [1–4]	3 [1–4]	<0.01
LKW to arrival (hour)	4.3 [1.6–10.9]	4.6 [1.6–11.5]	3.0 [1.4–8.2]	0.01
IV thrombolysis	117 (10.8%)	115 (12.3%)	2 (1.3%)	<0.01
LKW to IVT (hour)	2.4 [1.6–3.3]	2.5 [1.6–3.4]	2.2 [1.4–3.0]	0.73
Vascular risk factor				
Hypertension	666 (61.5%)	589 (63.1%)	77 (51.7%)	0.01
Diabetes	315 (29.1%)	269 (28.8%)	46 (30.9%)	0.68
Dyslipidemia	276 (25.5%)	246 (26.3%)	30 (20.1%)	0.13
Smoking	412 (38.0%)	356 (38.1%)	56 (37.6%)	0.97
Atrial fibrillation	304 (28.1%)	254 (27.2%)	50 (33.6%)	0.13
Imaging findings				
Baseline ASPECTS	10 [9–10]	10 [9–10]	10 [8–10]	0.48
Collateral grade, baseline				0.051
Poor (0, 1)	71 (6.9%)	55 (6.3%)	16 (11.0%)	
Intermediate (2, 3)	380 (37.1%)	322 (36.6%)	58 (40.0%)	
Good (4, 5)	573 (56.0%)	502 (57.1%)	71 (49.0%)	
END not related to EVT	207 (19.1%)	184 (19.7%)	23 (15.4%)	0.26
Treatment outcomes				
Any hemorrhage	172 (15.8%)	118 (12.6%)	54 (36.2%)	<0.01
Significant hemorrhage	51 (4.7%)	31 (3.3%)	20 (13.4%)	<0.01
mRS 0-1 at 3 months	576 (53.8%)	504 (54.5%)	72 (49.0%)	0.24
mRS 0-2 at 3 months	766 (71.5%)	668 (72.3%)	98 (66.7%)	0.19
Death up to 3 months	36 (3.4%)	30 (3.2%)	6 (4.1%)	0.78

Early neurological deterioration (END) is neurological deterioration before the initiation of EVT or in medically treated patients. Significant hemorrhage was defined as parenchymal hemorrhage type 2 (PH2) hemorrhagic transformation and class II of Heidelberg Bleeding Classification (23).

### Effectiveness of endovascular recanalization treatment

EVT was performed in 149 (14%) patients with a median of 5.9 h [IQR 3.6–12.3 h] from LKW. Patients who received EVT were younger, arrived earlier, had intracranial ICA or M1 segment MCA occlusion rather than extracranial ICA or M2 occlusion at baseline and had a history of hypertension. Moreover, IV thrombolysis was given in only two patients (1%) in the EVT group, while 115 (12%) patients were in the medically managed group. Patients who received EVT were more likely to have a significant hemorrhage but not excellent functional recovery at 3 months (mRS 0–1; Table 1).

When using regression models incorporating propensity scores with 1:1 matching, EVT was not associated with better functional recovery 3 months after stroke. However, EVT increased the odds of having significant hemorrhage (PS-matched OR, 4.51 [1.59–12.80]) or any hemorrhage (PS-matched OR, 3.17 [95% CI, 1.76–5.69]; Table 2). The results from the unadjusted logistic regression model and inverse probability weighting model are provided in Supplementary Figure III.

**Table 2:** Effectiveness and safety of EVT for mild LVO.

**Outcome**	**Multivariable**	**Propensity-score**
**indices**	**logistic model**	**matched model**
mRS 0–1 at 3 months	0.85 [0.57–1.28]	0.99 [0.63–1.54]
mRS 0–2 at 3 months	0.68 [0.44–1.05]	0.93 [0.57–1.51]
Overall distribution of mRS	0.84 [0.60–1.18]	0.83 [0.35–1.96]
Mortality at 3 months	0.95 [0.35–2.56]	1.27 [0.33–4.94]
Any hemorrhage	3.62 [2.30–5.69]	3.17 [1.76–5.69]
Significant hemorrhage	4.09 [2.06–8.11]	4.51 [1.59–12.80]

**Figure 1:**
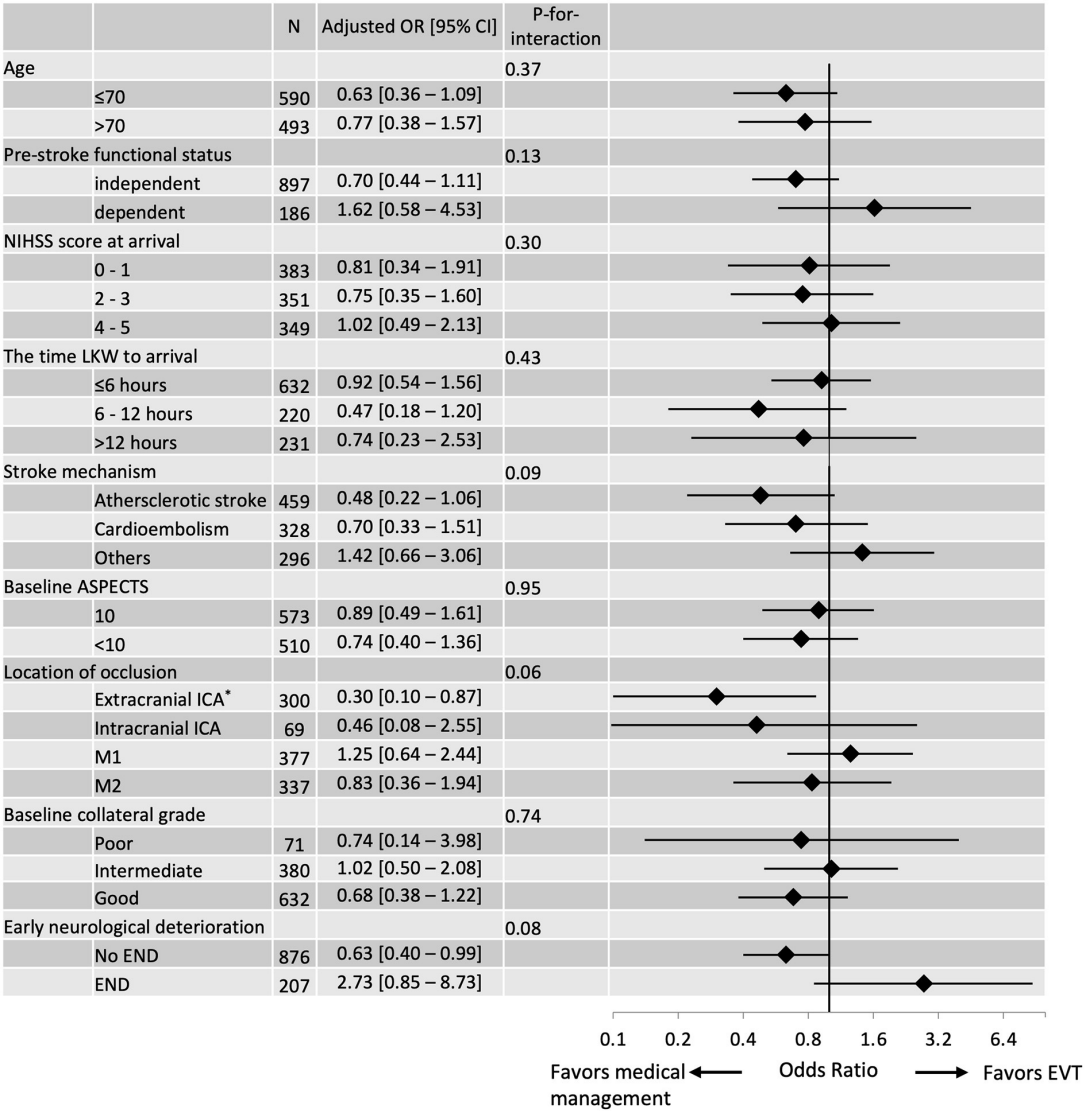
Subgroup analyses of EVT effectiveness were assessed using 90-day mRS 0–1 and stratified by selected baseline characteristics. The vertical line signifies the null point (OR 1.00). Adjusted ORs and 95% CIs were calculated using multivariable logistic regression models without applying the propensity score. *Extracranial ICA includes tandem lesions.

Subgroup analyses showed that the effectiveness of EVT was potentially modified by occlusion location (*P*-for-interaction, 0.06), underlying stroke mechanism (*P*-for-interaction, 0.09) and the occurrence of END prior to EVT (*P*-for-interaction, 0.08; Figure 1). Multivariable logistic regression models adjusted for baseline demographics, vascular risk factors, location of the occlusion, baseline collaterals, ASPECTS and preceding END showed that the odds of having a 90-day mRS score 0–1 by EVT decreased in patients with baseline extracranial ICA occlusion (adjusted OR 0.30 [95% CI, 0.10–0.87]), patients with atherosclerotic stroke (adjusted OR 0.48 [95% CI, 0.22–1.06] and patients without END (adjusted OR 0.63 [95% CI, 0.40–0.99]). There was no difference in the proportion of patients who received EVT achieving 90-day mRS 0–1 among those who had END prior to EVT (45.5%) vs. those without preceding END (49.6%; Supplementary Data I).

### Early neurological deterioration

**Figure 2:**
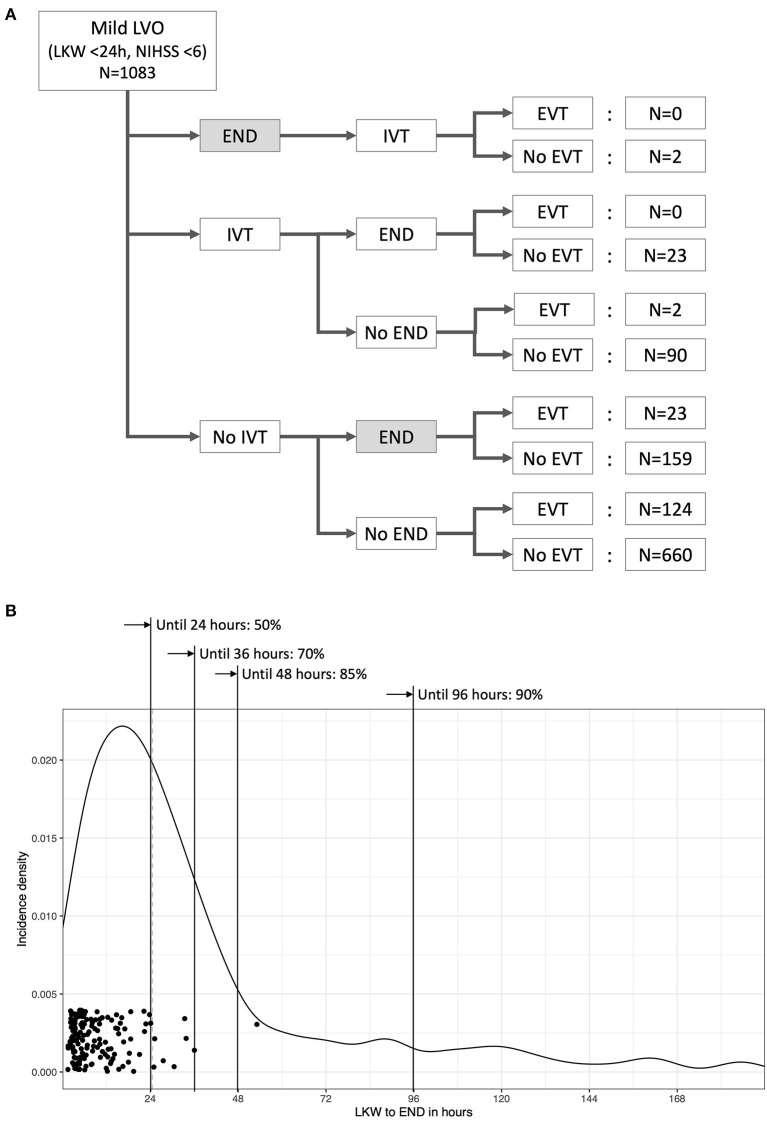
Temporal profile of early neurological deterioration (END) related to recanalization treatments for acute LVO patients with mild neurological deficits. Only ENDs captured before the initiation of EVT were counted for the present study. Of 207 ENDs (19% of all patients) that occurred before the initiation of EVT, 23 developed after intravenous thrombolysis. Five ENDs were symptomatic hemorrhages, one END occurred after intravenous thrombolysis and four ENDs occurred without recanalization treatment **(A)**. The median time from the last known well (LKW) to END was 24.5 h, and 85% of ENDs developed within 48 h from the time LKW. Dots represent the time of arterial puncture for EVT **(B)**.

END before initiation of EVT or in medically treated patients occurred in 207 (19%) patients (Figure 2A), and the median [IQR] duration between LKW time and END reporting was 24.5 h [13.5–41.9]. The incidence rate [95% CI] of END was 1.41 [1.23–1.62] per 100 person-hours, and 85% of ENDs occurred within 48 h from the LKW (Figure 2B). The NIHSS score after the occurrence of END was a median of 7 (4–10), which increased by a median of 4 (2–8) from baseline. Patients with END were more likely to have a higher baseline NIHSS score, a history of hypertension and extracranial ICA occlusion on baseline imaging. Patients with END had a higher proportion of unfavorable imaging and clinical outcomes (Table 3 and Supplementary Figure II). Mild END (NIHSS score increase <4) occurred in 97 (9.0%) subjects, four of whom had EVT; severe END (NIHSS score increase ≥4) developed in 109 (10.1%) patients, 19 (17.4%) of whom received EVT (Supplementary Data I).

**Table 3:** Comparison of acute LVO patients with low NIHSS scores (<6) by early neurological deterioration (END).

**Variables**	**END (–)**	**END (+)**	**P-for-**
	**(n, 876)**	**(n, 207)**	**difference**
Demographics			
Age (yrs, SD)	66.8 ± 13.1	68.9 ± 12.9	0.04
Male sex (*n*, %)	543 (62.0%)	126 (60.9%)	0.83
Prestroke dependency (*n*, %)	152 (18.3%)	34 (17.4%)	0.86
Stroke information			
Baseline NIHSS score (median, IQR)	2 [1–4]	3 [1–4]	0.03
TIA as an index stroke (*n*, %)	71 (8.1%)	2 (1.0%)	<0.01
Stroke mechanism (*n*, %)			0.50
Large artery atherosclerosis	300 (37.3%)	86 (42.0%)	
Cardioembolism	269 (33.4%)	59 (28.8%)	
Other determined etiology	39 (4.8%)	8 (3.9%)	
Undetermined etiology	197 (24.5%)	52 (25.4%)	
Occluded artery (*n*, %)			<0.01
Extracranial ICA (without tandem occlusions)	167 (19.1%)	56 (27.1%)	
Intracranial ICA	55 (6.3%)	14 (6.8%)	
M1	308 (35.2%)	69 (33.0%)	
M2 or distal MCA	297 (33.9%)	40 (19.3%)	
Tandem occlusion	49 (5.6%)	28 (13.5%)	
LKW to arrival in hours (median [IQR])	4.2 [1.6–10.9]	4.8 [1.6–11.0]	0.83
IV thrombolysis (*n*, %)	92 (10.5%)	25 (12.1%)	0.60
LKW to IV thrombolysis in hours (median [IQR])	2.5 [1.6–3.4]	1.8 [1.4–2.9]	0.09
Endovascular treatment (*n*, %)	126 (14.4%)	23 (11.1%)	0.26
LKW to groin puncture in hors (median [IQR])	5.5 [3.5–11.9]	8.2 [5.8–13.5]	0.08
Vascular risk factors (*n*, %)			
Hypertension	518 (59.1%)	148 (71.5%)	<0.01
Diabetes	245 (28.0%)	70 (33.8%)	0.11
Dyslipidemia	218 (24.9%)	58 (28.0%)	0.40
Smoking	333 (38.0%)	79 (38.2%)	0.99
Atrial fibrillation	246 (28.1%)	58 (28.0%)	0.99
Baseline imaging ratings			
ASPECTS (median [IQR])	10 (9,10)	9 (8-10)	0.12
Advanced WMH (*n*, %)	235 (27.3%)	68 (33.7%)	0.09
Old infarction, all (*n*, %)	296 (34.4%)	78 (38.6%)	0.30
CMB, all (*n*, %)	97 (11.3%)	20 (9.9%)	0.67
Collateral grade (*n*, %)			0.76
poor (0, 1)	55 (6.7%)	16 (8.1%)	
Intermediate (2, 3)	307 (37.1%)	72 (37.1%)	
Good (4, 5)	465 (56.2%)	108 (54.8%)	
Stroke outcomes			
Any hemorrhages on the follow-up image (*n*, %)	131 (15.0%)	41 (19.8%)	0.11
Significant hemorrhage	32 (3.7%)	19 (9.2%)	<0.01
Duration of hospital stay in days (median [IQR])	6.4 [4.6–9.4]	9.6 [6.3–15.5]	<0.01
mRS 0–1 at 3 months (*n*, %)	521 (60.0%)	55 (27.1%)	<0.01
mRS 0–2 at 3 months (*n*, %)	675 (77.8%)	91 (44.8%)	<0.01
Death at 3 months (*n*, %)	21 (2.4%)	15 (7.4%)	<0.01

^*^Early neurological deterioration (END) is neurological deterioration before EVT initiation or in medically treated patients. Categorical variables are summarized as frequencies (percentages), and continuous variables are summarized as the mean ± SD or median [IQR_25_ - IQR_75_]. Significant hemorrhage was defined as PH2 hemorrhagic transformation and HBC class II (23).

The time from LKW to END occurrence was shorter in patients who had EVT (median 6.7 h [IQR, 4.0–12.2] when compared to a median of 26.2 h (16–39), [41–44] in those who were treated (P-for-difference, <0.01). The NIHSS at END occurrence was a median of 9 (7–13) in those who received EVT and a median of 7 (4–10) in medically managed patients (P-for-difference, 0.27). In the multivariable logistic regression model in which the location of occlusion was mutually exclusive, the presence of extracranial ICA occlusion (adjusted OR 1.72, 95% CI [1.09–2.73]), presence of tandem occlusion (adjusted OR, 2.79 [1.57–4.95]), history of hypertension (adjusted OR, 1.49 [1.03–2.14]) and a higher NIHSS (adjusted OR 1.12 per 1-point increase, [1.02–1.23]) were significantly associated with higher odds of END occurrence (for full results, see Supplementary Table III).

## Discussion

In this large nationwide registry-based study of 1,083 acute LVO patients with low NIHSS scores, early neurological deterioration (END) occurred in approximately 20% of patients, mostly within 48 h from the time LKW. EVT in these patients may potentially result in increased hemorrhagic complications without an overall clinical benefit. However, the potential benefits vs. risks associated with EVT are likely modified by occlusion location, underlying stroke etiology and proceeding END.

Clinical decision-making in patients with acute ischemic stroke due to LVOs presenting with mild stroke symptoms is challenging. The mild nature of symptoms indicates that the risks of invasive treatments, such as EVT, need to be balanced against the risks of neurological deterioration as part of natural history. Physicians have therefore relied on observational studies to provide necessary evidence on this topic. The present study, with its large sample size, prospectively collected data from a nationwide acute stroke registry and high-quality imaging characterization and outcome ascertainment, adds substantially to the current body of literature and strength of evidence (28). The patient demographics and prevalence of END in the present study were similar to those of previously published studies from North America and Europe (4, 12, 13, 29).

However, data on when patients with acute LVOs and mild stroke worsen neurologically, i.e., have END, are conflicting. In a previous report on 32 patients from a single center, END developed after a median of 5.2 h from arrival with a prehospital delay of a median of 10.5 h (7). In an analysis of 347 IV thrombolysis-treated patients, 48% of ENDs occurred within 2 h after thrombolysis (13). In comparison, Park et al. reported that 61% of ENDs developed within 48 h from the time LKW according to their analysis of all subjects from a nationwide registry (22). The present study demonstrated that END was likely to occur within 48 h from LKW in 85% of patients. Interestingly, >50% of ENDs occurs 24 h or more after LKW. Therefore, these patients are less likely to be offered EVT after END based on current EVT practice guidelines, thus attesting to the need to predict END occurrence early after stroke onset (30, 31). The efficacy of EVT in these delayed progressive ischemic stroke patients beyond the conventional “time window” should be investigated.

In the present study, the administration of EVT increased the odds of hemorrhagic complications without any comparable increase in the proportion of patients achieving good clinical outcomes at 90 days. Therefore, these results add to the body of literature that advocates caution in offering EVT to such patients outside of ongoing clinical trials (4, 28, 32). The possibility that the effect of EVT may be modified by the occurrence of END prior to EVT administration is intriguing. From our subgroup analysis, mild LVO patients without END did not benefit from EVT. Thus, waiting for END to occur and then offering EVT is one way of altering the analysis of risks and benefits with EVT in favor of that therapy. However, this option also indicates that patients may only be offered EVT late when the potential risks of therapy are higher and the potential benefits are lower (16). Although we have reported benefits with EVT in patients presenting even beyond 24 hours (33), more data will be needed before such therapy is offered to patients with worsening clinical symptoms beyond 24 h from LKW. An alternative approach may be an assessment of the risk of END. The present study showed that patients with higher baseline NIHSS scores, a history of hypertension and the presence of extracranial ICA occlusion or tandem occlusion on baseline imaging are at higher risk of END. A risk score that identifies patients at high risk for END may help in determining the best candidates for EVT from mild LVO patients (13, 30).

Detailed imaging evaluation at baseline was a strength of the present study. In contrast to expectations, we did not find any association among baseline collateral status, the occurrence of END and effect of EVT, which may be because these patients with LVOs and mild symptoms invariably tend to have good collaterals (56% in the present study). As long as the collateral circulation provides sufficient cerebral perfusion to the ischemic area, invasive treatment may be delayed based on the premise that new leptomeningeal collaterals may develop after ischemia (34–36). However, these leptomeningeal collaterals are tenuous vessels, indicating that even subtle changes in cerebral perfusion pressure may alter hemodynamics to the extent that may cause END (37, 38). A more detailed analysis of cerebral circulation over time will be needed to understand the complex pathophysiology of leptomeningeal collaterals and how they affect cerebral blood flow over time in these patients.

There are ongoing prospective studies evaluating the clinical benefit of endovascular recanalization for acute LVO patients with mild neurological severity. “Endovascular Therapy for low NIHSS Ischemic Strokes (ENDOLOW)” from North America (NCT04167527) and “Minor Stroke Therapy Evaluation (MOSTE)” from Europe (NCT03796468) are two randomized clinical trials expected to prove the efficacy of EVT. “Mild Acute Ischemic Stroke With Large Vessel Occlusion (MISTWAVE)” is a prospective registry recruiting EVT cases with ICA, M1, M2 or basilar artery occlusion who had NIHSS scores <6 at baseline (NCT03731351). Stronger evidence from large high-quality datasets is needed to help physicians make the best treatment decisions in these patients until those prospective results will be available in a few years.

The present study had limitations. Data were obtained from patients predominantly of East Asian ethnicity who have a higher prevalence of intracranial stenosis (39). Differences in stroke etiology and hemorrhagic tendency due to ethnicity of patients may affect the extrapolation of these results to other populations. Practice differences, such as higher utilization of MRI for follow-up, may affect the relative prevalence of outcomes, such as hemorrhage, in the present study. Diagnostic imaging acquisition parameters and treatment strategies for acute LVO patients with mild neurological deficits also varied by hospital (Supplementary Table II). The use of propensity score matching and regression analysis likely mitigated some of this variability.

## Conclusion

Among acute LVO cases presenting with low NIHSS scores of ≤5 points, one-fifth of patients developed END, and 85% of patients developed END within 48 h of the LKW time. EVT was associated with increased hemorrhagic complications but no functional improvement even after balancing the baseline characteristics. The effectiveness of EVT was modified by stroke mechanisms, location of the occlusion and preceding END. The results of the present study did not permit general treatment recommendations for acute LVO patients with mild neurological deficits. Treatment decisions for the use of EVT for mild LVO patients require a prudent judgment based on the individual risk of stroke progression, expected benefit from the treatment and potential risk of complications, such as hemorrhagic transformation. Ongoing clinical trials will further inform this challenging clinical decision.

## Author's note

The STROBE statement is provided as Supplementary Data III.

## Data availability statement

The raw data supporting the conclusions of this article will be made available by the authors, without undue reservation.

## Ethics statement

The studies involving human participants were reviewed and approved by Institutional Review Board of the Seoul National University Bundang Hospital [B-2007-622-105]. The patients/participants provided their written informed consent to participate in this study.

## Author contributions

BeK: conception and design of the study. BuK, M-KH, JiK, JuK, K-JL, J-MP, KK, SL, J-KC, D-HK, J-HJ, TP, S-SP, KL, JunL, JuneL, K-SH, Y-JC, H-KP, B-CL, K-HY, M-SO, D-EK, W-SR, K-HC, JC, J-GK, J-HK, W-JK, D-IS, KY, S-IS, J-HH, CK, S-HL, and H-JB: acquisition of data. BeK, JY, JH, BuK, CKK, JGK, J-TK, HP, and SB: image analysis. BuK and BM: drafting and revision of the manuscript. All authors critical intellectual contribution for revision and approval of manuscript.

## Funding

This research was supported by a fund (2020ER620200#) by the Research Fund of Korea Centers for Disease Control and Prevention.

## Conflict of interest

The authors declare that the research was conducted in the absence of any commercial or financial relationships that could be construed as a potential conflict of interest.

## Publisher's note

All claims expressed in this article are solely those of the authors and do not necessarily represent those of their affiliated organizations, or those of the publisher, the editors and the reviewers. Any product that may be evaluated in this article, or claim that may be made by its manufacturer, is not guaranteed or endorsed by the publisher.
