# Supplementary material for: Effectiveness and safety of EVT in patients with acute LVO and low NIHSS
Source: Front Neurol. 2022 Aug 5;13:955725. doi: 10.3389/fneur.2022.955725 (PMC9389111; doi:10.3389/fneur.2022.955725)
Supplement: Supplementary file 1 [file Data_Sheet_1.PDF]

Online-only supplement

Effectiveness and Safety of EVT in patients with acute LVO and low NIHSS

Beom Joon Kim, Bijoy K. Menon, Joonsang Yoo, et al.

List of items

Supplemental Method I. Study flow chart
Supplemental Method II. Inter-rater agreement of image readings
Supplemental Method III. Standard Operation Procedure of Central Image Lab and Evaluation of Images
Supplemental Method IV. Statistical analysis plan
Supplemental Method V. Estimation of the propensity score for EVT and balancing

Supplemental Figure I. Distribution of mRS three months after stroke
Supplemental Figure II. Distribution of mRS by early neurological deterioration and treatment strategies
Supplemental Figure III. Effectiveness and safety of EVT for mild LVO patients

Supplemental Table I. Prevalence of missing information
Supplemental Table II. The disparity of treatment strategy for acute LVO patients with mild neurological deficits by hospitals
Supplemental Table III. Unadjusted and multivariable logistic regression models of variables associated with early neurological deterioration

Supplemental Data I. Details of early neurological deteriorations not related to the endovascular recanalization treatment
Supplemental Data II. Clinical Research Collaboration for Stroke in Korea investigators
Supplemental Data III. STROBE Statement

Supplemental Method I. Study flow chart

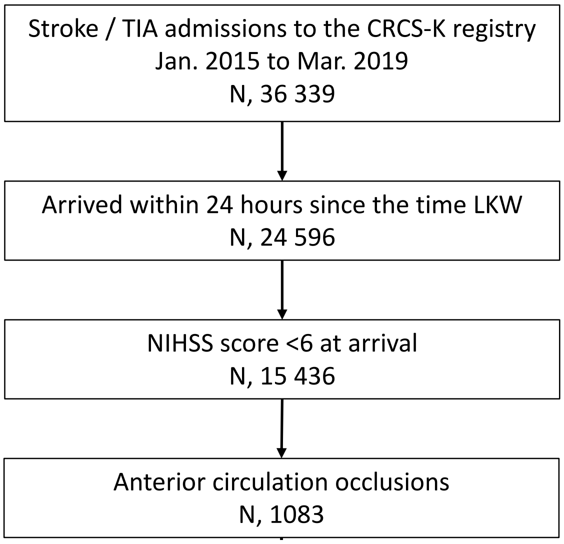

Supplemental Method II. Inter-rater agreements image readings

	Image modality	intraclass correlation coefficient
ASPECTS, left hemisphere	CT	0.94 [0.93 - 0.96]
ASPECTS, right hemisphere	CT	0.91 [0.89 - 0.93]
Collateral scores	CT	0.80 [0.68 - 0.88]
Hemorrhage	CT	0.93 [0.91 - 0.94]
ASPECTS, left hemisphere	MR	0.80 [0.76 - 0.83]
ASPECTS, right hemisphere	MR	0.87 [0.85 - 0.90]
White matter hyperintensities	MR	0.88 [0.85 - 0.91]
Old infarction, deep structures	MR	0.77 [0.72 - 0.81]
Old infarction, cortex	MR	0.80 [0.76 - 0.83]
Cerebral microbleeds, on GRE	MR	0.61 [0.52 - 0.68]
Cerebral microbleeds, on SWI	MR	0.85 [0.73 - 0.92]
Collateral scores	MR	0.77 [0.71 - 0.82]
Hemorrhage	MR	0.88 [0.86 - 0.90]
Collateral scores (ASITN/SIR)	Angiography	0.80 [0.68 - 0.88]
eTICI score	Angiography	0.83 [0.60 - 0.94]

Supplemental Method III. Standard Operation Procedure of Central Image Lab and Evaluation of Images

Multicenter collaboration of acute ischemic stroke with minor neurological deficit and emergent large vessel occlusion: Collateral status and endovascular treatment in the real-world clinical practice (mild ELVO): Standard operation procedure of central image lab

First Created on March 13, 2020
Last modified on August 23, 2020

mildELVO

Correspondence to Beom Joon Kim, MD.PhD
Department of Neurology and Cerebrovascular Center,
Seoul National University Bundang Hospital
82 Gumi-ro 173 beon-gil, Bundang-gu, Seongnam-si, Gyeonggi-do, 13620, Republic of Korea
E-mail: Kim.BJ.Stroke@gmail.com
Phone: +82-31-787-7468
Fax: +82-31-787-4567

The current document is written in Korean and English. Should there be any disagreement in the interpretation, the document in Korean will have priority.

1. Objective of the document

The current document, the standard operation procedure of the central image lab, is composed to define the image collection, storage, backup, evaluation, and construction of database procedures for the multicenter collaboration of acute ischemic stroke with minor neurological deficit and emergent large vessel occlusion (mild ELVO) project.

2. Overview and objective of the project

2.1. Overview of the mild ELVO project
Based on the multicenter, nationwide, prospective Clinical Research Collaboration for Stroke in Korea (CRCS-K) registry, the mild ELVO project constructs and analyzes a retrospective database of clinical and image data for emergent large vessel occlusion (LVO) patients with low NIHSS score.

2.2. Objective of the mild ELVO project
Primary research objectives
From the emergent LVO patients with low NIHSS score, the mild ELVO project will analyze 1) collateral status and associated clinical factors, 2) incidence of early neurological deterioration (END) and defined a high-risk group of END, and 3) the effectiveness of endovascular recanalization (EVT) in the real-world clinical practice out of a clinical trial.

3. List of Central Image Lab members

Beom Joon Kim (vascular neurologist, Seoul National University Bundang Hospital), Joonsang Yoo (interventional neurologist, Yong-In Severance Hospital), Hyungjong Park (interventional neurologist, Dongsan Medical Center, Keimyung University), Joon-Tae Kim (vascular neurology, Chonnam National University Hospital), Jae Gook Kim (interventional neurology, Eulji University Hospital), Chi Kyung Kim (vascular neurologist, Korea University Guro Hospital), Jung Hoon Han (interventional neurologist, Korea University Guro Hospital), Bum Joon Kim (vascular neurologist, Asan Medical Center), Sung Hyun Baik (Interventional radiologist, Seoul National University Bundang Hospital)

4. Collection of image data

4.1. Selection of target population
Source DB: CRCS-K DB (1903)_20200106.mdb
IN-01 (n, 15436): admitted after Jan/01/2015, baseline NIHSS score <6, and arrived ≤24 hours from the time last known well

- WHERE ((([initial-K].arrival)>=#1/1/2015#) AND ((([arrival]-[lnt])*24)<=24) AND (([initial-K].ini_nih)<6));

IN-02 (n, 1366): recorded to have anterior circulation occlusion in the database

- WHERE ((([extend 1].a_mca)=1) AND (([extend 1].a_mca_s)=3)) OR ((([extend 1].a_inica)=1) AND (([extend 1].a_inica_s)=3)) OR ((([extend 1].a_exica)=1) AND (([extend 1].a_exica_s)=3)) OR ((([extend 1].a_cca)=1) AND (([extend 1].a_cca_s)=3));

Hospital (anonymized)	Number of the target population
NE	33
DE	44
DA	180
SB	185
SM	31
SH	16
YN	108
IP	61
HS	19
CN	320
DI	24
CB	49
US	86
JN	64
KMD	101
CH	5

4.2. Collection of image data

An anonymized list of the target population will be sent to participating hospitals from the central image lab.

- Anonymized fields; [Uni_num], [male], [age], [lnt], [fat], [arrival], [dis_d]

Patients will be identified in the participating hospitals from the encrypted database.
All the neuroimages are taken between 1 week before the [arrival] and [dis_d].

- Brain CT, CT angiography, CT perfusion (including source image)
- Brain MRI, MR angiography, diffusion-weighted image, MR perfusion (including source image)
- Cerebral angiography (regardless of diagnostic or therapeutic angiography)

Images should be saved in the anonymized Dicom format, with the folder name of [Uni_num] and subsequent image examination folders.

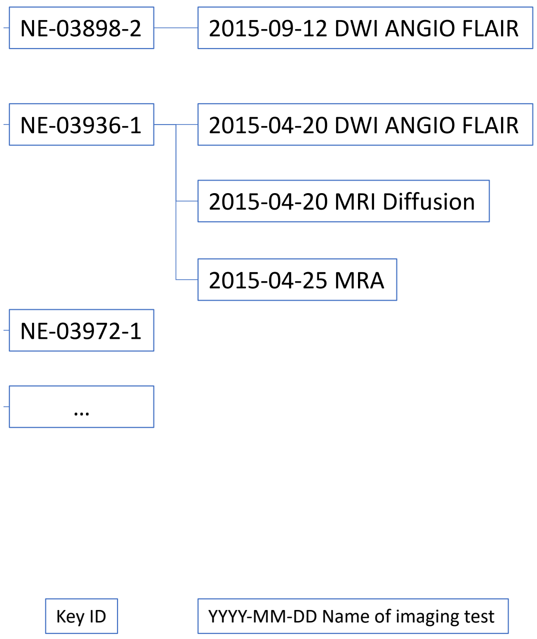

After saving image data from the first five subjects, all the images should be sent to the central image lab. Image collection procedures will resume after confirmation from the central image lab.

5. Anonymization of individual private information

Each participating hospital performs anonymization procedures according to the standard procedure of the center. Central image lab anonymizes all the collected image data again with OsiriX MD software.

6. Storage of image data and backup

6.1. Storage of image data

Central image lab securely stores the collected images, with the folder structure of hospital - individual admissions - individual examinations.

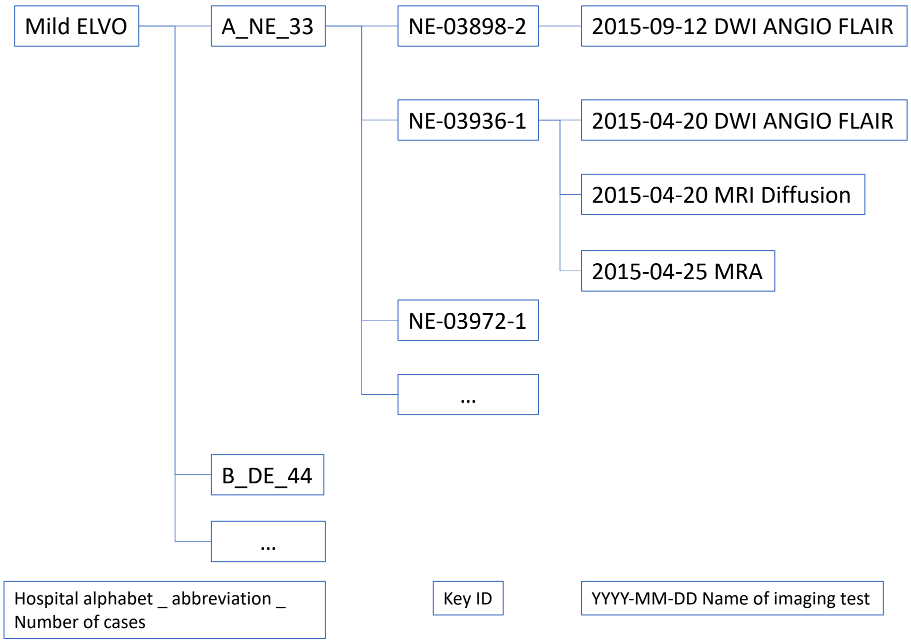

6.2. Back up of stored data

The central image lab duplicates all the images to a separate hard disk drive and stores them in a different place.

7. Construction of image database

7.1. Image DB by hospitals

The image database for the OsirixMD will be constructed by each of the individual hospitals.

7.2. Secondary anonymization and generation of KeyID

Using the anonymization tool from the OsiriX MD, individual private information in the Dicom files will be deleted, such as PatientAge (0010,1010), PatientID (0010,0020), PatientName (0010,0010), PatientBirthDate (0010,0030).

KeyID ([Uni_num] of the CRCS-K registry) will be recorded in the PatientID (0010, 0020).

8. Database design

8.1. DB structure

Web database for image rating has two key identifiers.

- [uni_num]; Key identifier for individual admission and linkage to the CRCS-K clinical database
- [image_ID]; Key identifier for individual image examinations, based on the hospital record

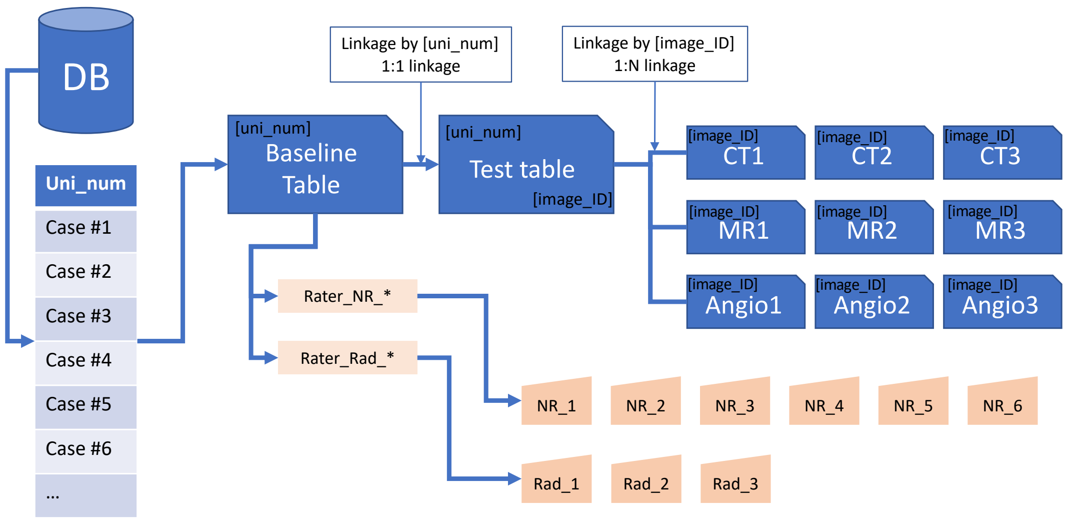

DB structure

Unique [uni_num] for one admission
Unique [image_ID] for one order of image test

Scalable design to raters and image tests

Database tables include

- Baseline table: information for each admission
- Key ID: [uni_num]
- Test table: information for image work-ups
- Key ID: [uni_num] - linkage to test table
- Key ID: [image_ID] - linkage to individual examination tables
  - 1:N matching for [uni_num] and [image_ID]

- CT1, CT2, CT3 table: table for rating CT images
- MR1, MR2, MR3 table: table for rating MR images
- Angio1, Angio2, Angio3 table: table for rating angiography images

8.2. DB dictionary for web database
- redacted

9. Image rating

9.1. Image reading method
The readings described below performs as consensus readings of more than two readers (vascular neurologists and interventional neurologists). Discrepancies between raters are determined by an independent neuroradiologist (SHB) and discussions. All the angiography images are additionally read by interventional neuroradiologists (CGJ or SHB). Before commencing the image reading, all the raters evaluated 50 individual cases, and consensus of the reading was reached with neuroradiologists' supervision.
Images are reviewed under appropriate conditions (dimmed light, multi-monitor setup, windowing tools).

ASPECT score

- 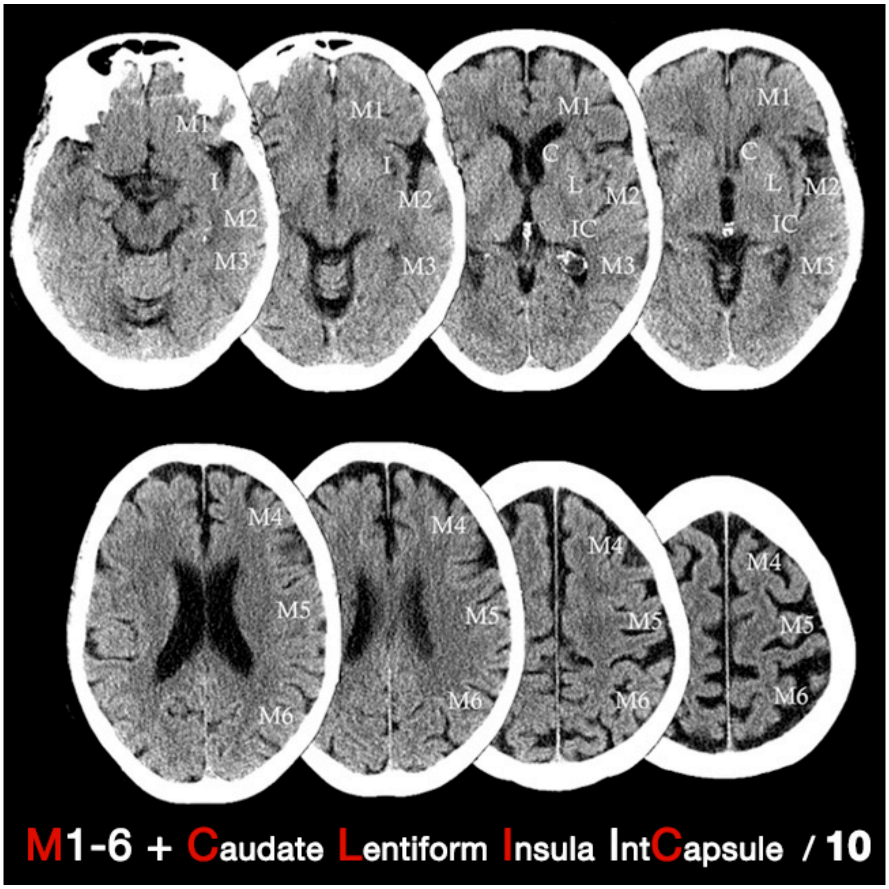

- Five mm reconstructed axial unenhanced head CT images are available. The readers are blinded to the clinical data but have access to the other images such as angiography or MR.
- ASPECTS: The ASPECTS are scored on NCCT images to assess the extent of early ischemic changes. Areas with early ischemic changes (relative hypodensity, loss of grey-white matter differentiation, and effacement of sulci) are considered as affected, and the regions affected were reported explicitly.
- Affected side: The affected side (side of early ischemic changes and/or side of hyperdense or unsure hyperdense vessel sign) is reported. In the case of early ischemic changes in the anterior circulation, it is reported whether the right or left side was affected. In the case of supratentorial and cerebellar posterior circulation changes, the affected side (right or left) is reported as well. In the case of brainstem early ischemic changes, no side is reported.
- ASPECTS are recorded based on the standard template, and the readers are recommended to use their discretion in the difference of acquisition axes between CT and MR. ASPECTS scores are counted when at least one-third of the affected region has DWI lesions.
- Barber PA, et al. Validity and reliability of a quantitative computed tomography score in predicting the outcome of hyperacute stroke before thrombolytic therapy. ASPECTS Study Group. Alberta Stroke Programme Early CT Score. Lancet. 2000;355:1670-1674.

sCTA collateral and TOF-MRA collateral

Pial arterial filling score within the symptomatic ischemic territory

Score	Original score	Summary	Tan score (for comparison)
5	When compared with asymptomatic contralateral hemisphere, there is <u>increased or normal</u> prominence and extent of pial vessels within the ischemic territory in the symptomatic hemisphere	≥100% than normal	3
4	When compared with the asymptomatic contralateral hemisphere, there is <u>slightly reduced</u> prominence and extent of pial vessels within the ischemic territory in the symptomatic hemisphere	90% - 100% than normal	2
3	When compared with the asymptomatic contralateral hemisphere, there is <u>moderately reduced</u> prominence and extent of pial vessels within the ischemic territory in the symptomatic hemisphere	50 - 90% than normal	2
2	When compared with the asymptomatic contralateral hemisphere, there is <u>decreased prominence and extent and regions with no vessels</u> within the ischemic territory in the symptomatic hemisphere	≤50% and regions without vessels	1
1	When compared with the asymptomatic contralateral hemisphere, there <u>are just a few vessels visible</u> within the ischemic territory in the occluded vascular territory	minimal vessels	1

0	When compared with the asymptomatic contralateral hemisphere, there are no vessels visible within the ischemic territory in the symptomatic hemisphere	0%	1

- Menon BK, et al. Multiphase CT Angiography: A New Tool for the Imaging Triage of Patients with Acute Ischemic Stroke. Radiology. 2015; 275:510-520.
- Tan YL, et al. CT Angiography Clot Burden Score and Collateral Score: Correlation with Clinical and Radiologic Outcomes in Acute Middle Cerebral Artery Infarct. Am J Neuroradiol. 2009;30:525-531.

Pial arterial filling score by multiphase CT angiography

- Rating criteria: phase delay / extent / prominence

Score	Original score	Summary
5	When compared with asymptomatic contralateral hemisphere, there is no delay and normal or increased prominence of pial vessels/normal extent within the ischemic territory in the symptomatic hemisphere	normal
4	When compared with asymptomatic contralateral hemisphere, there is a delay of one phase in filling in of peripheral vessels, but prominent and extent is the same	One phase delay
3	When compared with asymptomatic contralateral hemisphere, there is a delay of two phases in filling in of peripheral vessels, or there is a one-phase delay and significantly reduced number of vessels in the ischemic territory	Two phases delay and normal extent<br>OR<br>One phase delay and reduced number of vessels
2	When compared with asymptomatic contralateral hemisphere, there is a delay of two phases in filling in of peripheral vessels and decreased prominence and extent, or a one-phase delay and some ischemic regions with no vessels	Two phases delay with decreased prominence/extent<br>OR<br>One delay with some regions with no vessels
1	When compared with asymptomatic contralateral hemisphere, there are just a few vessels visible in any phase within the occluded vascular territory	minimal vessels
0	When compared with asymptomatic contralateral hemisphere, there are no vessels visible in any phase within the ischemic vascular territory	No vessels

- Menon BK, et al. Multiphase CT Angiography: A New Tool for the Imaging Triage of Patients with Acute Ischemic Stroke. Radiology. 2015; 275:510-520.

Location of occlusion

- Primary occlusion: The primary occlusion was defined as the most proximal occlusion that subsequently affects the largest brain parenchyma volume. The most proximal end of the clot was decisive for the clot localization (e.g., a clot that extends from the ICA in the M1 and M2 was reported as an ICA occlusion). The vessels were noted as follows:
  - ICA: Intra- (C2 to C7 of ICA) or extracranial ICA (C1). The beginning was defined as the carotid bifurcation and the end as the ICA bifurcation.
  - M1: Begins distal to the ICA bifurcation (distal to C7) and ends at the main bifurcation in the inferior and superior M2 branch/at the trifurcation in case of a trifurcation.
  - M2: M2 segment extending from the MCA main and ends at the circular sulcus of the insula
  - A1: Starts at the ICA bifurcation, ends at the origin of the anterior communicating artery.
  - A2: Starts at the origin of the anterior communicating artery, ends at the genu of the corpus callosum
  - P1: Origins at the terminal bifurcation of the basilar artery ends at the origin of the posterior communicating artery
  - P2: Starts at the origin of the posterior communicating artery and courses around the mesencephalon ends when the vessel enters the quadrigeminal cistern
  - Basilar artery: Starts at the junction of the vertebral arteries and ends at the basilar bifurcation
  - Readers are recommended to use their discretion in reading variant anatomy cases.

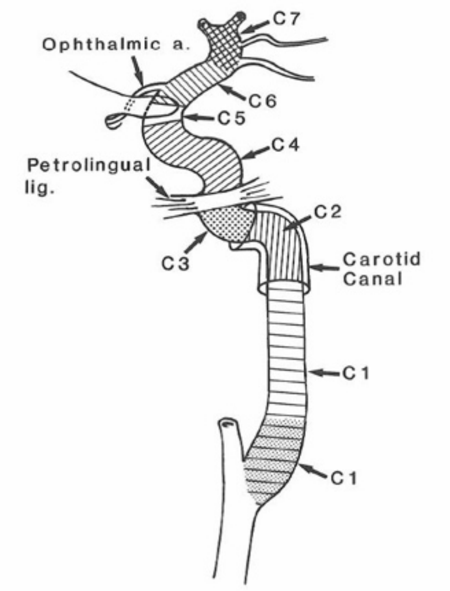

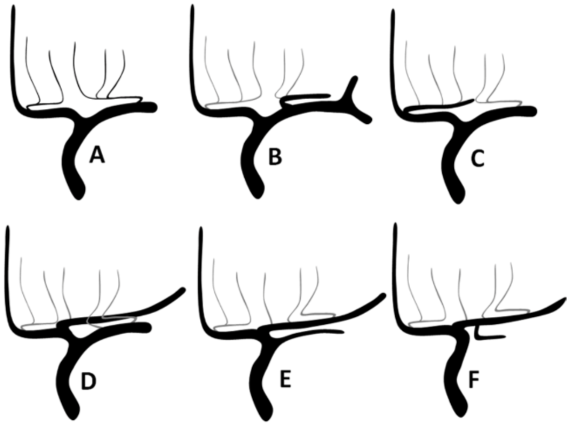

- Shapiro M, et al. Neuroanatomy of the middle cerebral artery: implications for thrombectomy. J Neurointerv Surg. 2020
- Osborn AG. Diagnostic cerebral angiography. 1999

White matter hyperintensity of presumably vascular origin

- Signal abnormality of variable size in the white matter that shows the following characteristics: hyperintensity on T2-weighted images such as fluid-attenuated inversion recovery, without cavitation (signal different from CSF). Lesions in the subcortical grey matter or brainstem are not included in this category unless explicitly stated.

Fazekas grading

- 0, No lesions (including symmetrical, well-defined caps or bands)
- 1, focal lesions
- 2, beginning confluence of lesions
- 3, diffuse involvement of the entire region/with or without the involvement of U fibers

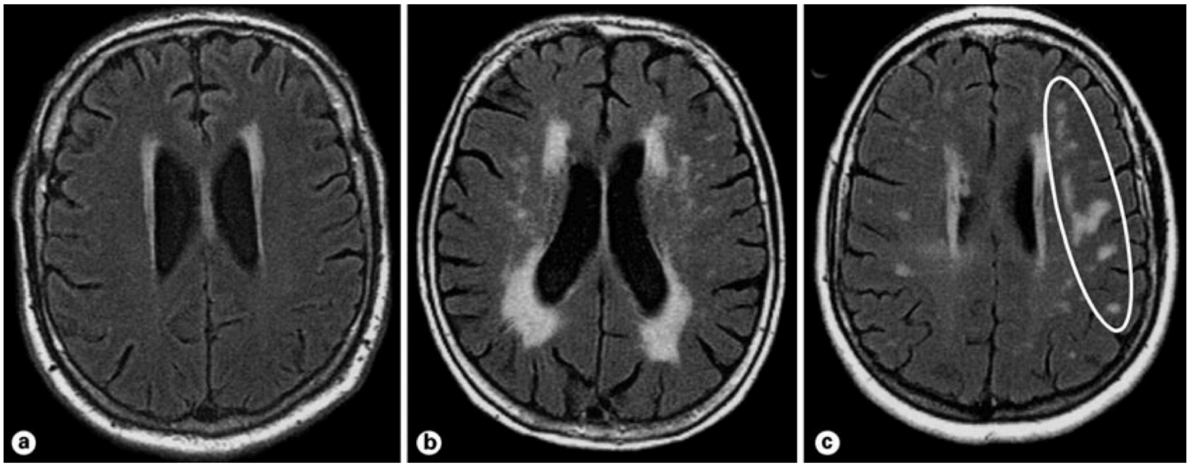

Fig. 3. FLAIR scans show WML at different locations: (**a**) rim of periventricular hyperintensity, (**b**) periventricular hyperintensity extending into the deep white matter, and (**c**) deep WML separated from periventricular signal changes as indicated by the ellipsoid.

- Wardlaw et al. Neuroimaging standards for research into small vessel disease and its contribution to ageing and neurodegeneration. Lancet Neurol. 2013
- Fazekas F, et al. CT and MRI rating of white matter lesions. Cerebrovasc Dis. 2003;13:31-36.

Lacune of presumably vascular origin

- A round or ovoid, subcortical, fluid-filled cavity (signal similar to CSF) of between 3 mm and about 15 mm in diameter, consistent with a previous acute small subcortical infarct or hemorrhage in the territory of one perforating arteriole.
- Location of old infarction is categorized as cortical (cortical gray matter or centrum semiovale) and deep (caudate nucleus, putamen, external capsule, internal capsule, or thalamus) structure
- Wardlaw et al. Neuroimaging standards for research into small vessel disease and its contribution to ageing and neurodegeneration. Lancet Neurol. 2013

Cerebral microbleeds

- Small (generally 2-5 mm in diameter, but sometimes up to 10 mm) areas of a signal void with associated blooming seen on T2*-weighted MRI or other sequences that are sensitive to susceptibility effects
- Cerebral microbleeds are read separately on GRE and SWI sequences. The type of image sequences is also recorded.
- Wardlaw et al. Neuroimaging standards for research into small vessel disease and its contribution to ageing and neurodegeneration. Lancet Neurol. 2013

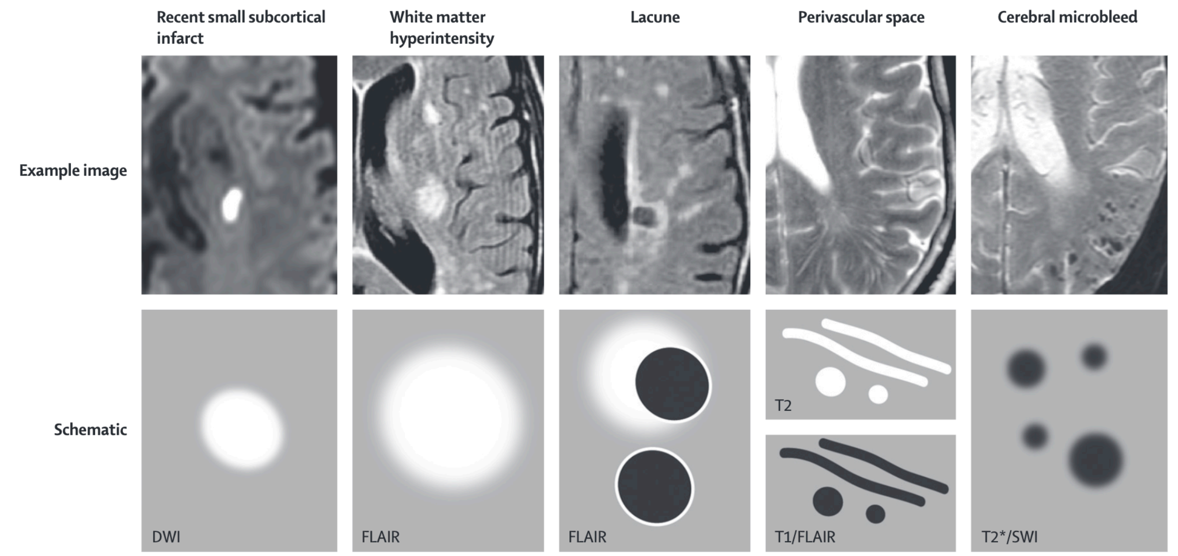

	Recent small subcortical infarct	White matter hyperintensity	Lacune	Perivascular space	Cerebral microbleed
**Usual diameter**	≤20 mm	Variable	3–15 mm	≤2 mm	≤10 mm
**Comment**	Best identified on DWI	Located in white matter	Usually have hyperintense rim	Most linear without hyperintense rim	Detected on GRE seq., round or ovoid, blooming
**DWI**	↑	↔	↔/(↓)	↔	↔
**FLAIR**	↑	↑	↓	↓	↔
**T2**	↑	↑	↑	↑	↔
**T1**	↓	↔/(↓)	↓	↓	↔
**T2*-weighted GRE**	↔	↑	↔ (↓ if haemorrhage)	↔	↓↓

↑ Increased signal ↓ Decreased signal ↔ Iso-intense signal

***Figure 2:* MRI findings for lesions related to small vessel disease**
Shows examples (upper) and schematic representation (middle) of MRI features for changes related to small vessel disease, with a summary of imaging characteristics (lower) for individual lesions. DWI=diffusion-weighted imaging. FLAIR=fluid-attenuated inversion recovery. SWI=susceptibility-weighted imaging. GRE=gradient-recalled echo.

Angiography collateral

- ASITN/SIR grading
    - 0=no collaterals visible to the ischemic site
    - 1=slow collaterals to the periphery of the ischemic site with the persistence of some of the defect
    - 2=rapid collaterals to the periphery of the ischemic site with the persistence of some of the defect and only a portion of the ischemic territory
    - 3=collaterals with slow but complete angiographic blood flow of the ischemic bed by late venous phase
    - 4=complete and rapid collateral blood flow to the vascular bed in the entire ischemic territory by retrograde perfusion
- Zaidat OO, et al. Stroke. 2013;44:2650-2663

eTICI score

- 0, No reperfusion
- 1, 1 (reduction in thrombus w/o filling of distal arterial branches)

- 2, 2a (0 - 49% of territory)
- 3, 2b50 (50 - 66% of territory)
- 4, 2b67 (67 - 89% of territory)
- 5, 2c (extensive reperfusion in 90-99%)
- 6, 3 (complete or full reperfusion)

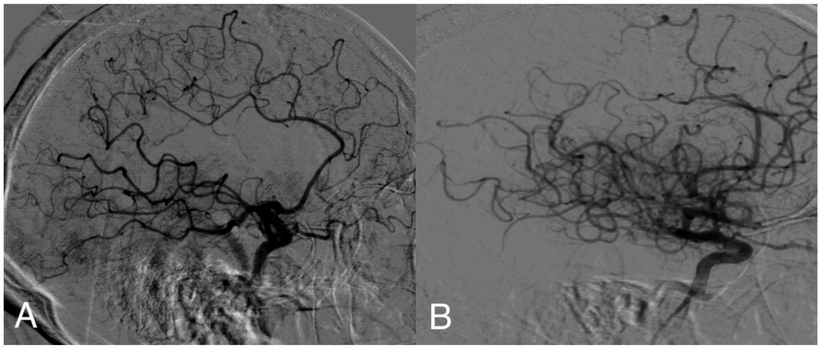

Figure 1 Angiography of final reperfusion of the middle cerebral artery territory showing (A) eTICI 2b50 (50–66%) versus (B) eTICI 2b67 (67–89%).

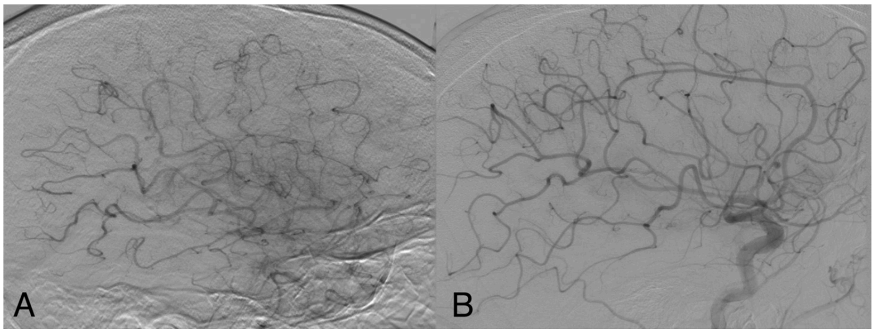

Figure 2 Angiography of final reperfusion of the middle cerebral artery territory showing (A) eTICI 2c (90–99%) versus (B) eTICI 3 (100%).

- Liebeskind DS, et al. eTICI reperfusion: defining success in endovascular stroke therapy. J Neurointervt Surg. 2019;11:433-438.

9.2. Procedures for image rating

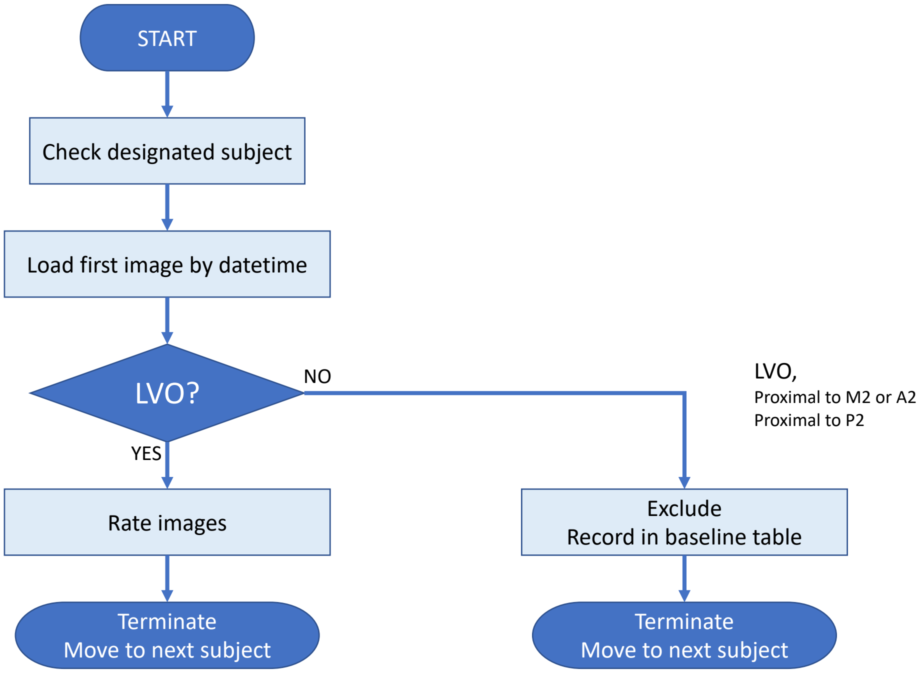

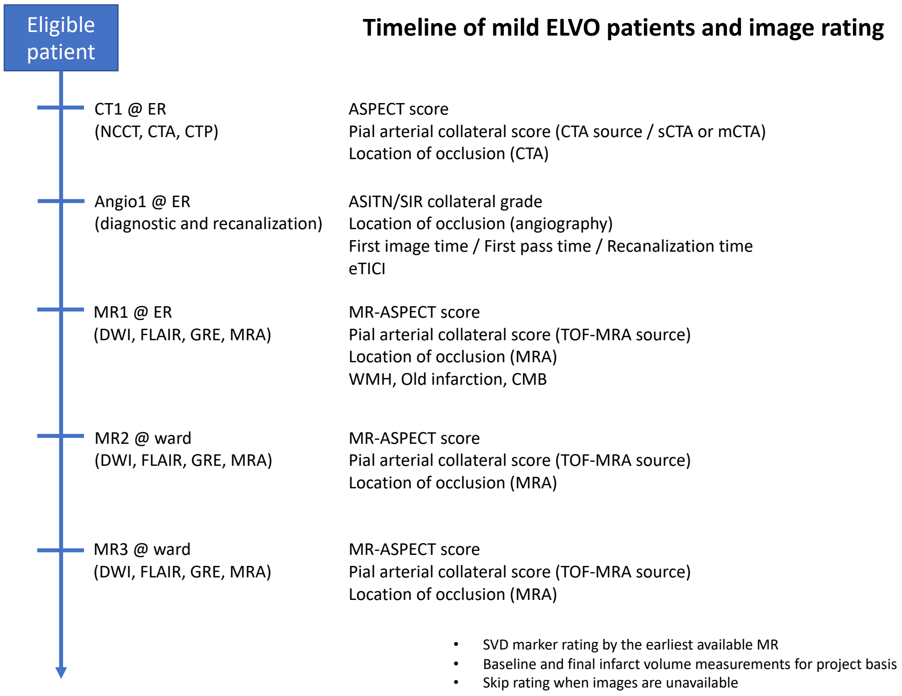

Supplemental Method IV. Statistical analysis plan

Multicenter collaboration of acute ischemic stroke with minor neurological deficit and emergent large vessel occlusion: Collateral status and endovascular treatment in the real-world clinical practice (mild ELVO): Statistical Analysis Plan

Correspondence to Beom Joon Kim, MD.PhD
Department of Neurology and Cerebrovascular Center,
Seoul National University Bundang Hospital
82 Gumi-ro 173 beon-gil, Bundang-gu, Seongnam-si, Gyeonggi-do, 13620, Republic of Korea
E-mail: Kim.BJ.Stroke@gmail.com
Phone: +82-31-787-7468
Fax: +82-31-787-4567

The current document is written in Korean and English. Should there be any disagreement in the interpretation, the document in Korean will have priority.

Introduction

1. Background and rationale

- Currently, mild ELVO patients who had baseline NIHSS equal to or less than 5-point are not eligible for endovascular recanalization treatment, according to the most up-to-date guidelines. [Powers. Stroke. 2019]
- Series of publications in 2018 and 2019 suggested that EVT for the mild ELVO is feasible and efficacious. [Sarraj, Stroke 2018; Nagel, Stroke 2018; Goyal N, JAMA Neurol 2020]
- The ENDOLOW trial is about to start near future to test the efficacy of EVT in anterior circulation ELVO patients with baseline NIHSS ≤5-point. [NCT04167527]
    - Without a published design paper, the inclusion criteria of the ENDOLOW trial did not specify image modalities and salvageable tissues. Trialst will naturally randomize mild ELVO patients harboring a substantial amount of "target mismatch," but a smaller sample size (175 vs. 175) will not be sufficient for further detailed post hoc analyses.
- Treatment decision regarding EVT for mild ELVO involves careful consideration about the expected benefit of the treatment; EVT will save AIS patients from a disabling stroke in the near future which does not happen at the time of decision.
- Thus, optimal selection of patients with mild ELVO who are at high risk of early neurological deterioration will be as important as the efficacy of EVT itself.
    - We also need to consider the heterogeneity of the hospital setting of caring for mild ELVO patients; perfusion images may not be available in all the primary stroke centers, at which mild stroke patients will first visit.

2. Objectives

- Among the anterior circulation ELVO patients with baseline NIHSS ≤5-point;
    - To find out the frequency of adequate versus poor collateral circulation and associated clinical factors
    - To find out a high-risk subgroup for early neurological deterioration (END) and associated image and clinical factors
    - To examine the effectiveness of EVT among the high-risk subgroup
- Secondary analyses will include (but not confined to)
    - the efficacy of IV tPA in mild ELVO patients
    - the validity of different image modalities (sCTA, mCTA, perfusion images) in selecting high-risk subgroups
    - the characteristics of END in mild ELVO
    - endovascular techniques and treatment device of mELVO; I-type occlusion, ICADs, distal embolization, and further items to be determined
    - Publication of secondary analysis results will be discussed in the steering committee.

Study methods

3. Study design

- This study is a retrospective cohort analysis based on an ongoing stroke registry in Korea, Clinical Research Collaboration for stroke in Korea (CRCS-K) registry. [Kim BJ. J Stroke. 2015] Imaging data were retrospectively collected and evaluated in the central image core lab. Details of the image core lab were described in a separate document.

- All the analyses will be performed in the superiority framework. Due to the retrospective nature, there will be no interim statistical analysis nor stopping guidance.
- Statistical analyses will be started when the central image core lab completes its evaluation.

4. Power considerations

- There is a controversy in the effectiveness of EVT for acute LVO patients with mild neurological deficit. Recently published well-designed researches reported non-significant associations between EVT and functional recovery. [Goyal. JAMA Neurol. 2020; Sarraj. Stroke. 2018] However, previous studies had limited sample sizes with less than 200 to 400.
- Our dataset is expected to have more than 1000 cases presented with acute LVO with an NIHSS score ≤5. Therefore, we may present substantial real-world evidence regarding EEVT'seffectiveness as well as additional subgroup analyses to highlight the best candidates for EVT among mild LVO patients.

Statistical principles

5. Confidence intervals and P-values

- The statistical significance level will be set as a two-tailed P-value <0.05.
- Regression coefficients or odds ratio will be presented with 95% confidence intervals.
- Considering the retrospective, hypothesis-generating, and exploratory nature of the study, we will not consider the increased chance of a type I error after multiple testing.
- For P-values from interaction analyses, a P-value between ≥0.05 and <0.10 will be considered dubious significance and will emphasize the increased chance of a type I error. [Pan. Stat Med. 1997]

Study population

6. Source database and selection criteria

- The source dataset will be the CRCS-K database containing cases up to March 2019. The total number of recorded cases is 73,033 as of November 2019.
- There will be no screening procedures for the current study.
- There will be no excluded cases due to withdrawal or failure to follow up, as the current analysis is based on the established and audited database.

- Inclusion criteria
  - Admitted after January 01, 2015
  - Baseline NIHSS score ≤5
  - Arrived within 24 hours after the time last known well
  - Documented LVO in the anterior circulation
    - Extracranial ICA (with or without tandem occlusions in distal tributaries), intracranial ICA, M1 or M2 segment of MCA
    - The presence of LVO will be confirmed through the central imaging lab. Therefore, the analysis dataset will be finalized after the image reading.

- Exclusion criteria
    - No specific predetermined exclusion criteria will be set, considering the exploratory and observative nature of the study.

7. List of covariates including potential confounders

- Basic demographic variables
    - Age, sex, pre-stroke dependency (modified Rankin Scale)
- Stroke information
    - initial NIHSS score, the time last known well, the time first symptom/sign detection, the time of hospital arrival
    - Potential etiology of stroke
- Vascular risk factors (history)
    - hypertension, diabetes, dyslipidemia, smoking, and atrial fibrillation
- Laboratory information
    - white blood cell count, blood urea nitrogen, creatinine, hemoglobin, hematocrit, total cholesterol, triglyceride, high-density lipoprotein, low-density lipoprotein, hemoglobin A1c, prothrombin time, C-reactive protein, systolic blood pressure, diastolic blood pressure
- In-hospital stroke management and clinical course
    - intravenous thrombolysis, the time of injecting thrombolytics
    - endovascular treatment, the time of groin puncture, the time of recanalization
    - early neurological deterioration (END), the time of reporting END, potential etiology of END, NIHSS score at END
    - Date of discharge
- Stroke outcomes
    - modified Rankin Scale at three months after stroke
    - Recurrent stroke and mortality
- Imaging information
    - Arterial occlusion location
    - ASPECTS
    - Cerebral small vessel disease markers: cerebral white matter hyperintensities, old infarction, cerebral microbleeds
    - Collateral grade
    - Hemorrhagic transformation
    - ASITN/SIR collateral and eTICI score

Analysis

8. Outcome definitions

- The primary outcome of the study will be mRS 0 - 1 three months after stroke.
- Secondary effectiveness outcomes will include mRS 0 - 2 and the overall distribution of mRS three months after stroke.
- Secondary safety outcomes will include mortality up to three months after stroke, any hemorrhagic transformation, and significant hemorrhages.
    - Significant hemorrhage is defined as parenchymal hemorrhage type II and/or class II of the Heidelberg Bleeding Classification.

- For analyses of END, the occurrence of END before the EVT or in medically-treated patients will be the primary outcome.

9. Analysis methods

- Patients characteristics will be summarized using frequencies (percentages) for categorical variables, means ± standard deviations for continuous variables with normal distribution and medians [interquartile ranges] for continuous variables with non-normal distribution. Groupwise comparison will be performed using chi-square tests for categorical variables and t-test for continuous variables.
- For END analysis, a binary logistic regression model taking END as a dependent variable will be constructed, with covariates bivariate P-value <0.20 as potential predictors and confounders.
- For analyzing the effectiveness of EVT in the study population, the propensity score will be used as a primary statistical tool to adjust the imbalance between the EVT group and the medical management group.
    - A propensity score for receiving endovascular treatment (EVT) will be generated using age, sex, pre-stroke dependency (pre-stroke mRS ≥1), occlusion location, potential etiology of stroke (categorized into large artery atherosclerosis, cardioembolism, and other or undetermined etiologies for model stability), initial NIHSS score, the time from LKW to arrival, intravenous thrombolysis, hypertension, diabetes, dyslipidemia, smoking, atrial fibrillation, baseline collateral grade, and baseline ASPECTS, taking EVT as a dependent variable.
    - The primary method of balancing the EVT group and medical management group will be matching. Individual patients from the two treatment groups will be matched 1:1 with a caliber making of 0.2 without replacement through a genetic search algorithm which determines the optimal weight to give each covariate. [Sekhons. J Stat Software 2011]
    - The efficiency of balancing will be confirmed through the global chi-square test and histograms before and after matching. Standardized mean differences (SMD) from all the included covariates will be tabulated.
    - As sensitivity analyses, the inverse probability of having EVT will be calculated and used as weighting. SMDs from all the included covariates will be tabulated.
    - Covariates with SMDs of 0.20 or more after weighting will be further incorporated into the weighting model.
- As sensitivity analyses, unadjusted and multivariable binary or ordinal logistic regression models will be constructed for secondary effectiveness and safety outcome indices. Multivariable logistic regression models will take covariates whose bivariate P-values <0.20.

10. Missing data

- Considering the observational and exploratory nature of the current study, the main strategy of dealing with the missing data is the complete case analysis. However, if a clinically important confounder shows a prevalence of missing ≥10%, the multiple imputations will be considered.
    - Multiple imputations will be performed using five imputed datasets with a presumption of missing at random.
- Outcome variables, such as mRS and END, will not be imputed.
- The prevalence of the missing variables will be presented as supplemental data.

11. Additional analyses

- Subgroup analyses
  - Effectiveness of EVT will be additionally tested in subgroups of age (cut off points to be determined at a near point of a median), pre-stroke dependency, NIHSS score at arrival (0 - 1, 2 - 3, 4 - 5), the time from LKW to arrival (≤6 hours, 6 - 12 hours, >12 hours), etiology of stroke (large artery atherosclerosis, cardioembolism, and other or undetermined etiologies), ASPECTS (cut off points to be determined at a near point of a median or mode), occlusion location (extracranial ICA, intracranial ICA, M1 segment or M2 segment of MCA), baseline collateral grade (poor, intermediate and good) and early neurological deterioration (no END or END).
    - Additional subgroups may be added based on the patients' characteristics.
  - The significance of interactions between subgroup variables and EVT will be tested.
  - Subgroup analyses will take mRS 0 - 1 three months after stroke as a dependent variable and use binary logistic regression models without applying propensity score matching or weighting.
- Tables
  - Table 1. Baseline characteristics
  - Table 2. Bivariate tables by END
  - Table 3. Multivariable logistic regression analyses results for END
  - Table 4. Bivariate tables by EVT
  - Table 5. The effectiveness of EVT, including PS-matching, weighting, unadjusted logistic, and multivariable logistic regression models
  - Table 6. Subgroup analyses for the effectiveness of EVT

12. Statistical software

- The R Project for Statistical Computing will be used. (https://cran.r-project.org)

Supplemental Method V. Estimation of a propensity score and balancing between treatment groups

Propensity scores (PS) for having endovascular treatment (EVT) were generated using age, sex, prestroke dependency, location of the occlusion, stroke mechanism, baseline National Institute of Health stroke scale (NIHSS) score, the time from last known well to arrival, intravenous alteplase, hypertension, diabetes, dyslipidemia, smoking, atrial fibrillation, baseline collateral grade, and baseline Alberta stroke program early CT score (ASPECTS). The variances of included variables between treatment groups were significantly different (P-value <0.001 from overall chi-square test; refer to the histogram between treatment groups before PS matching).

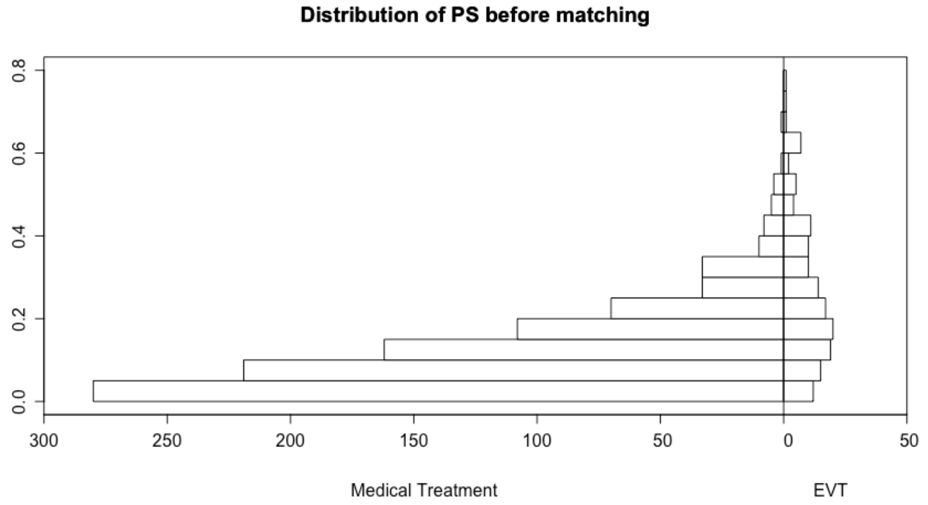

For matching, each EVT case was matched to one medical treatment control with a caliber making of 0.2 without replacement through a genetic search algorithm. [Sekhons. J Stat Software 2011] The variance between treatment groups after matching was not different (P-value 0.99 from overall chi-squared test; refer to the histogram between treatment groups after PS matching).

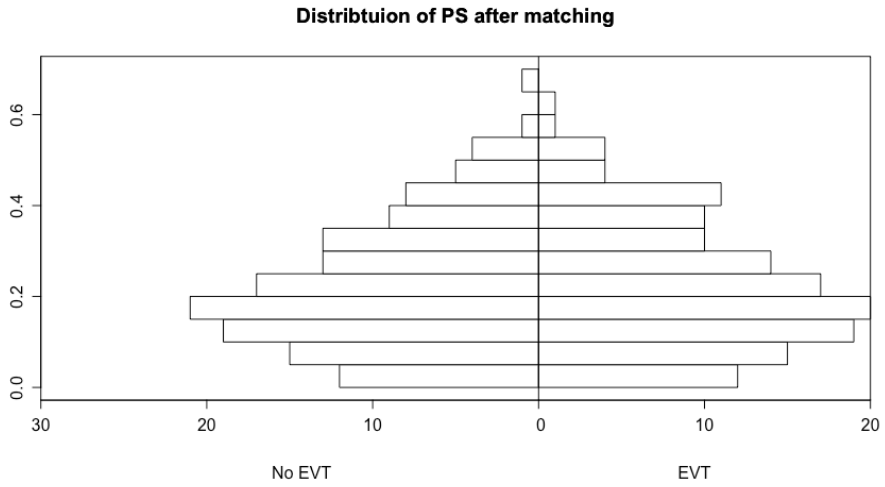

Inverse probabilities of EVT were calculated using the PS and weighted to individual subjects.

Standardized mean differences (SMD) of included variables in the raw dataset, matched dataset, and weighted dataset were calculated and presented in the table below.

	Unmatched			Matched			Weighted		
	0	1	SMD	0	1	SMD	0	1	SMD
n	934	149		138	138		1077.28	1400.84	
age (mean (SD))	67.62 (13.16)	64.80 (12.61)	0.219	65.75 (14.00)	65.62 (11.96)	0.011	67.26 (13.37)	66.91 (9.51)	0.03
male sex (%)	574 (61.5)	95 (63.8)	0.048	97 (70.3)	88 (63.8)	0.139	666.8 (61.9)	1057.5 (75.5)	0.296
prestroke dependency (%)	161 (17.2)	25 (16.8)	0.012	27 (19.6)	24 (17.4)	0.056	186.0 (17.3)	524.7 (37.5)	0.465
Occluded artery (%)			0.433			0.057			0.265
Extracranial ICA	203 (21.7)	20 (13.4)		19 (13.8)	20 (14.5)		222.7 (20.7)	274.1 (19.6)	
Intracranial ICA	51 (5.5)	18 (12.1)		16 (11.6)	14 (10.1)		68.4 (6.4)	88.9 (6.3)	
M1	308 (33.0)	69 (46.3)		63 (45.7)	62 (44.9)		371.9 (34.5)	338.1 (24.1)	
M2 or distal	301 (32.2)	36 (24.2)		34 (24.6)	36 (26.1)		336.7 (31.3)	585.7 (41.8)	
Tandem occlusion	71 (7.6)	6 (4.0)		6 (4.3)	6 (4.3)		77.6 (7.2)	114.1 (8.1)	
Stroke mechanism (%)			0.352			0.111			0.338
LAA	417 (44.6)	42 (28.2)		49 (35.5)	42 (30.4)		459.8 (42.7)	810.2 (57.8)	
CE	275 (29.4)	53 (35.6)		44 (31.9)	49 (35.5)		326.7 (30.3)	370.9 (26.5)	
ODE or UDE	242 (25.9)	54 (36.2)		45 (32.6)	47 (34.1)		290.8 (27.0)	219.8 (15.7)	
Baseline NIHSS (median [IQR])	2.00 [1.00, 4.00]	3.00 [2.00, 4.00]	0.362	3.00 [2.00, 5.00]	3.00 [1.00, 4.00]	0.09	2.00 [1.00, 4.00]	1.00 [1.00, 3.00]	0.287
LKW to arrival (median [IQR])	4.60 [1.65, 11.46]	3.05 [1.43, 8.15]	0.307	3.51 [1.72, 7.72]	3.21 [1.52, 8.62]	0.007	4.45 [1.64, 10.98]	4.32 [2.90, 10.72]	0.06
Intravenous thrombolysis (%)	115 (12.3)	2 (1.3)	0.446	3 (2.2)	2 (1.4)	0.054	117.0 (10.9)	324.4 (23.2)	0.332
Hypertension (%)	589 (63.1)	77 (51.7)	0.232	76 (55.1)	76 (55.1)	<0.001	667.0 (61.9)	680.3 (48.6)	0.271
Diabetes (%)	269 (28.8)	46 (30.9)	0.045	42 (30.4)	42 (30.4)	<0.001	311.7 (28.9)	286.3 (20.4)	0.198

Dyslipidemia (%)	246 (26.3)	30 (20.1)	0.147	34 (24.6)	29 (21.0)	0.086	275.7 (25.6)	280.6 (20.0)	0.133
Smoking (%)	356 (38.1)	56 (37.6)	0.011	57 (41.3)	50 (36.2)	0.104	408.5 (37.9)	696.1 (49.7)	0.239
Atrial fibrillation (%)	254 (27.2)	50 (33.6)	0.139	39 (28.3)	45 (32.6)	0.095	301.0 (27.9)	296.8 (21.2)	0.157
Baseline collaterals (%)			0.224			0.047			0.249
Poor	55 (5.9)	16 (10.7)		14 (10.1)	14 (10.1)		70.8 (6.6)	84.1 (6.0)	
Intermediate	322 (34.5)	58 (38.9)		50 (36.2)	53 (38.4)		376.7 (35.0)	658.8 (47.0)	
Good	557 (59.6)	75 (50.3)		74 (53.6)	71 (51.4)		629.7 (58.5)	657.9 (47.0)	
Baseline ASPECTS (mean (SD))	9.12 (1.28)	9.03 (1.35)	0.067	8.86 (1.35)	8.99 (1.38)	0.09	9.10 (1.30)	9.24 (1.19)	0.115

The numbers of included subjects in the weighted dataset were exaggerated due to the weighting procedure. Therefore, we calculated standard errors of the association between EVT and outcome indices using robust sandwich estimators to address the expansion.

All but age, sex and smoking achieved the desirable cut-off of SMD <0.1 in the matched dataset. But the covariates in the weighted dataset remained largely unbalanced after weighting (refer to the distribution of SMD below).

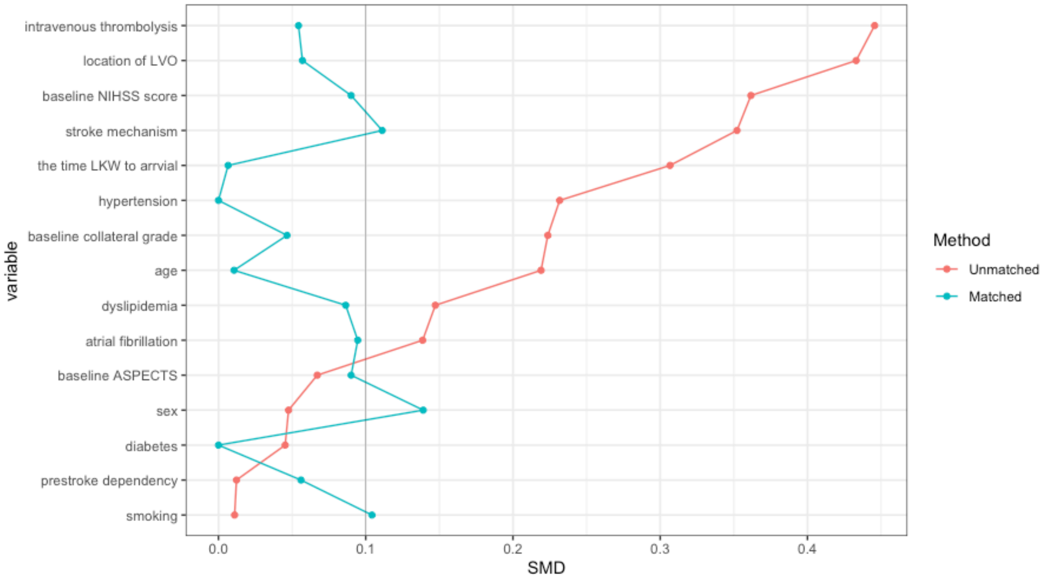

weighting vs. unmatched figure

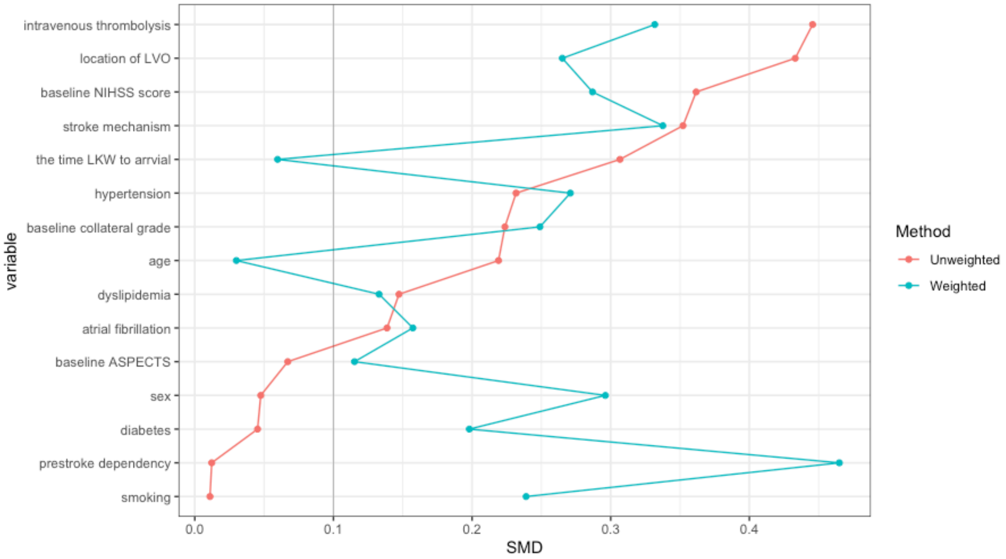

Here, we decided to incorporate covariates with a standardized mean difference of ≥ 0.20 after the weighting model. Further adjusted variables were intravenous thrombolysis, location of LVO, baseline NIHSS score, stroke mechanism, hypertension, baseline collateral grade, sex, prestroke dependency, and smoking.

Subgroup analyses were performed using binary logistic regression models taking mRS score 0 - 1 as a dependent variable and adjusted for age, sex, prestroke dependency, baseline NIHSS score, stroke mechanism, the time from LKW to arrival, intravenous thrombolysis, hypertension, diabetes, dyslipidemia, smoking, atrial fibrillation, baseline collateral grade and END not related to EVT.

Supplemental Figure I. Distribution of mRS three months after stroke

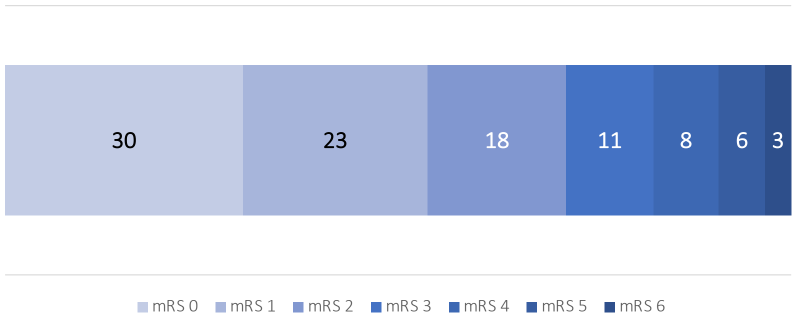

Supplemental Figure II. Distribution of mRS by early neurological deterioration and treatment strategies of mRS three months after stroke

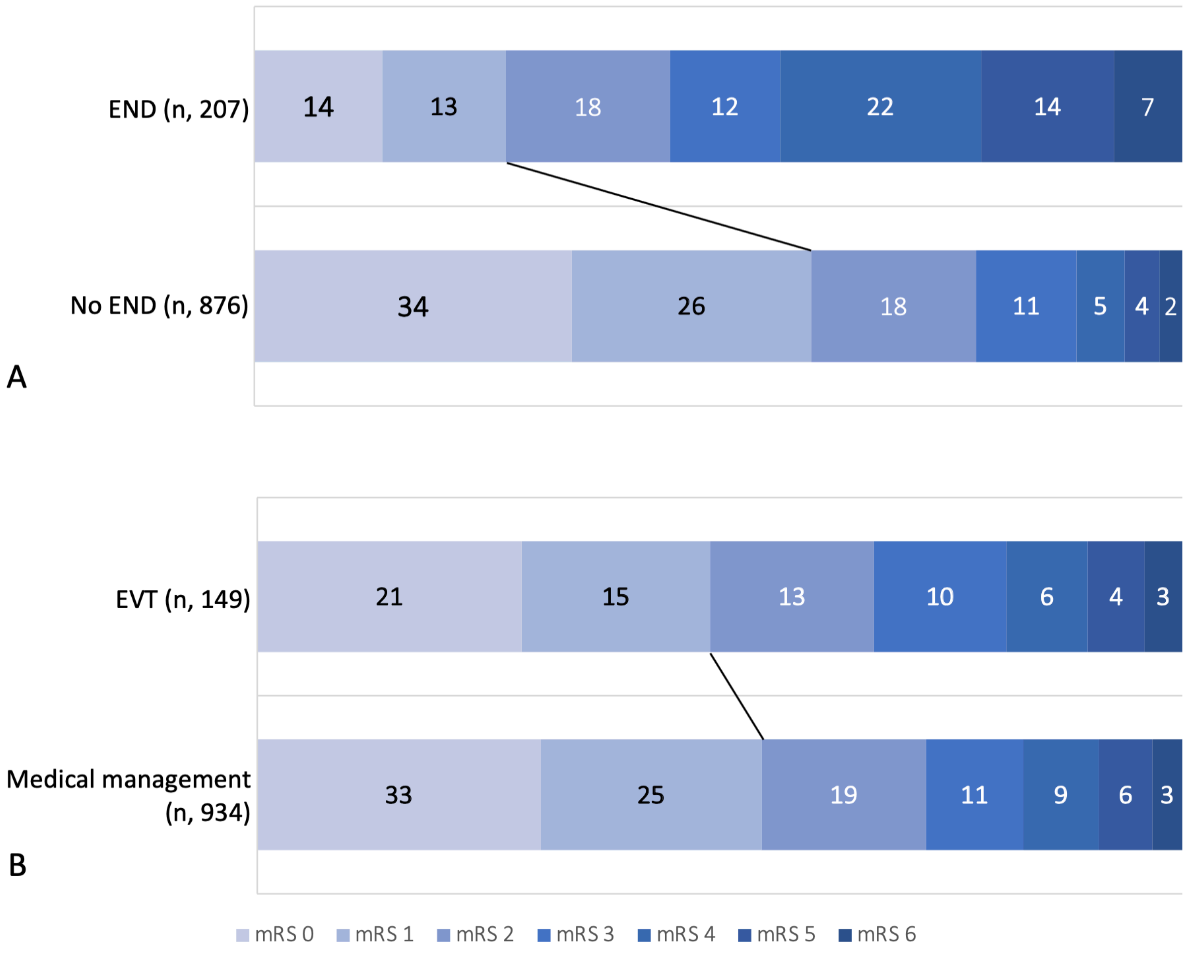

Distribution of mRS by early neurological deterioration (A) and treatment strategies (B). Early neurological deterioration (END) is neurological deterioration before initiation of EVT or in medically treated patients.

Supplemental Figure III. Effectiveness and safety of EVT for mild LVO patients

Functional outcomes and models		OR [95% CI]
mRS 0 - 1 at 3 months		
	Unadjusted logistic regression	0.80 [0.56 - 1.13]
	Multivariable logistic regression	0.85 [0.57 - 1.28]
	Matched set	0.99 [0.63 - 1.54]
	IPTW	0.73 [0.47 - 1.14]
mRS 0 - 2 at 3 months		
	Unadjusted logistic regression	0.77 [0.53 - 1.11]
	Multivariable logistic regression	0.68 [0.44 - 1.05]
	Matched set	0.93 [0.57 - 1.51]
	IPTW	0.68 [0.42 - 1.10]
Overall distribution of mRS		
	Unadjusted logistic regression	0.84 [0.61 - 1.14]
	Multivariable logistic regression	0.84 [0.60 - 1.18]
	Matched set	0.83 [0.35 - 1.96]
	IPTW	0.73 [0.43 - 1.22]

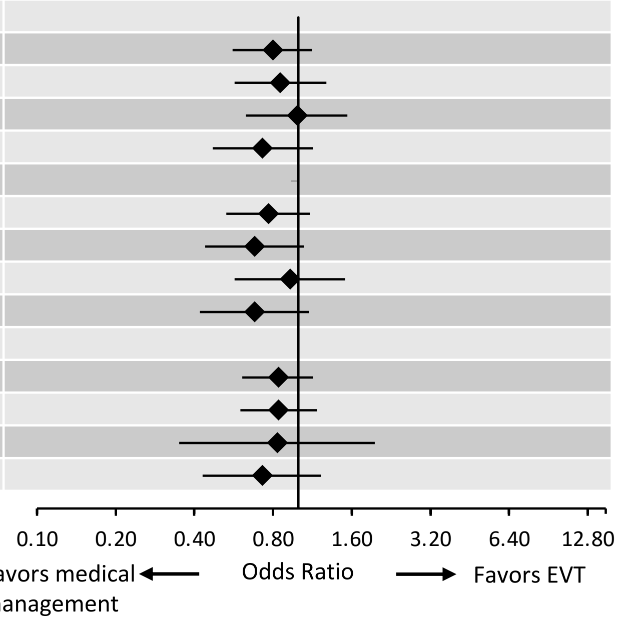

Safety outcomes and models		OR [95% CI]
Mortality at 3 months		
	Unadjusted logistic regression	1.27 [0.52 - 3.10]
	Multivariable logistic regression	0.95 [0.35 – 2.56]
	Matched set	1.27 [0.33 - 4.94]
	IPTW	0.36 [0.14 - 0.97]
Any hemorrhage		
	Unadjusted logistic regression	3.93 [2.67 - 5.78]
	Multivariable logistic regression	3.62 [2.30 - 5.69]
	Matched set	3.17 [1.76 - 5.69]
	IPTW	5.02 [2.94 - 8.57]
Significant hemorrhage		
	Unadjusted logistic regression	4.52 [2.50 – 8.16]
	Multivariable logistic regression	4.09 [2.06 - 8.11]
	Matched set	4.51 [1.59 - 12.80]
	IPTW	4.54 [2.05 - 10.07

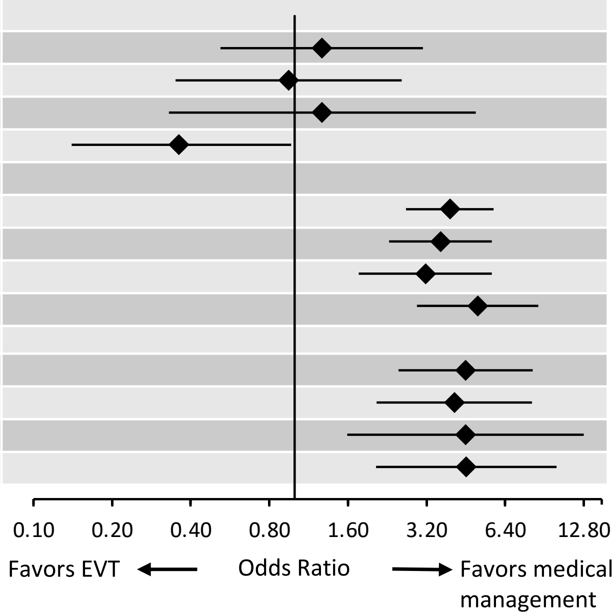

Supplemental Table I. Prevalence of missing information

Variable	Frequency (percentages)
sex	0
age	0
time indices for stroke (last known well, arrival, treatment initiation, and discharge)	0
Baseline NIHSS score	0
Functional status before the index stroke	57 (5.3%)
Etiology of stroke	0
Vascular risk factors (hypertension, diabetes, dyslipidemia, smoking, and atrial fibrillation)	0
Occlusion location	0
Systolic blood pressure	18 (1.7%)
Diastolic blood pressure	18 (1.7%)
Early neurological deterioration	0
Type	
modified Rankin Scale score at three months after stroke	12 (1.1%)
ASPECTS at baseline	5 (0.5%)
White matter hyperintensities, old infarction	21 (1.9%)
Cerebral microbleeds	20 (1.8%)
Collateral grades	59 (5.4%)
Hemorrhagic transformation	0

Supplemental Table II. The disparity of treatment strategy for acute LVO patients with mild neurological deficits by hospitals

Hospital	No IVT	IVT	Proportion of IVT (%)	No EVT	EVT	Proportion of EVT (%)
A	24	5	20.8	29	0	0.0
B	31	7	22.6	38	0	0.0
C	114	39	34.2	145	8	5.5
D	149	5	3.4	113	41	36.3
E	21	5	23.8	26	0	0.0
F	12	3	25.0	15	0	0.0
G	75	4	5.3	71	8	11.3
H	40	8	20.0	43	5	11.6
I	10	2	20.0	10	2	20.0
K	236	18	7.6	205	49	23.9
L	18	5	27.8	23	0	0.0
M	35	5	14.3	35	5	14.3
N	73	3	4.1	60	16	26.7
O	53	4	7.5	52	5	9.6
P	71	3	4.2	64	10	15.6
R	4	1	25.0	5	0	0.0

Supplemental Table III. Unadjusted and multivariable logistic regression models of variables associated with early neurological deterioration*

Variable	Unadjusted OR [95% CI]	Adjusted OR [95% CI]
Age (per 1-year)	1.01 [1.001 - 1.03]	1.00 [0.99 - 1.02]
Male	0.95 [0.70 - 1.30]	0.91 [0.64 - 1.30]
Occluded artery		
Extracranial ICA	1.50 [1.004 - 2.23]	1.72 [1.09 - 2.73]
Intracranial ICA	1.14 [0.60 - 2.16]	1.20 [0.61 - 2.37]
M1	reference	reference
M2	0.60 [0.39 - 0.92]	0.58 [0.37 - 0.92]
Tandem occlusion	2.55 [1.50 - 4.35]	2.79 [1.57 - 4.95]
NIHSS score (per score)	1.10 [1.01 - 1.20]	1.12 [1.02 - 1.23]
LKW to arrival (per hour)	1.00 [0.98 - 1.02]	0.99 [0.96 - 1.01]
Hypertension	1.73 [1.25 - 2.41]	1.49 [1.03 - 2.14]
Diabetes	1.32 [0.95 - 1.82]	1.13 [0.79 - 1.61]
Advanced WMH	1.35 [0.97 - 1.87]	1.05 [0.72 - 1.53]
Baseline ASPECTS (per 1-point decrease)	1.11 [0.996 - 1.24]	1.12 [0.99 - 1.27]
Collateral grade at baseline		
Poor	1.25 [0.69 - 2.27]	1.15 [0.59 - 2.25]
Intermediate	1.02 [0.74 - 1.42]	0.92 [0.63 - 1.32]
Good	reference	reference

ICA, internal carotid artery; NIHSS, National Institute of Health Stroke Scale; LKW, last known well; WMH, white matter hyperintensities; ASPECTS, Alberta Stroke Program Early Computed Tomography Score

* Early neurological deterioration (END) is neurological deterioration before EVT initiation or in medically treated patients.

Supplemental Data I. Details of early neurological deteriorations not related to the endovascular recanalization treatment

The total incidence of early neurological deterioration (END) occurred before the endovascular treatment (EVT) or in patients with medical treatment with or without intravenous thrombolysis was 207 (19.1%). Probable causes of the END tallied as following;

Probable causes of END	frequency (percentages)
Recurrent ischemic stroke	20 (9.8%)
Progression of previous ischemia	168 (81.2%)
Hemorrhagic transformation	5 (2.4%)
Unstable medical condition	6 (2.9%)
Undetermined	6 (2.9%)

The probable causes of END was not recorded in two (1.0%) cases.

The NIHSS score on the END was a median of 7 [interquartile range, 4 - 10], which increased by a median of 4 [2 - 8] from the baseline score.

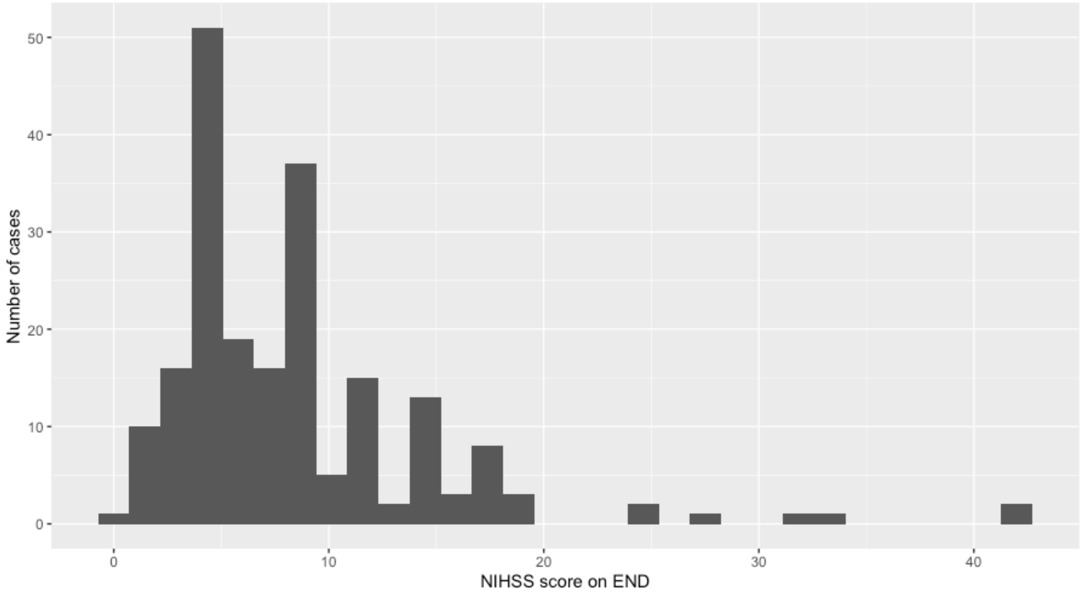

END was further categorized into mild END (NIHSS score increase <4) and severe END (NIHSS score increase ≥4), and the baseline clinical variables were distributed as follows:

Variables	No END (n=876)	mild END (n=97)	severe END (n=109)	P-for-difference
Demographics				
Age (yrs, SD)	66.8 ± 13.1	67.2 ± 13.5	70.5 ± 12.3	0.01
Male sex (n, %)	543 (62.0%)	60 (61.9%)	65 (59.6%)	0.89

Pre-stroke dependency (n, %)	152 (18.3%)	14 (14.9%)	20 (20.0%)	0.63
Stroke information				
Baseline NIHSS score (median, IQR)	2 [1 - 4]	3 [2 - 4]	2 [1 - 4]	<0.01
TIA as an index stroke (n, %)	71 (8.1%)	1 (1.0%)	1 (0.9%)	<0.01
Stroke mechanism (n, %)				0.14
Large artery atherosclerosis	300 (37.3%)	42 (43.8%)	43 (39.8%)	
Cardioembolism	269 (33.4%)	20 (20.8%)	39 (36.1%)	
Other determined etiology	39 (4.8%)	3 (3.1%)	5 (4.6%)	
Undetermined etiology	197 (24.5%)	31 (32.3%)	21 (19.4%)	
Occluded artery (n, %)				<0.01
Extracranial ICA (without tandem occlusions)	167 (19.1%)	25 (25.8%)	31 (28.4%)	
Intracranial ICA	55 (6.3%)	7 (7.2%)	7 (6.4%)	
M1	308 (35.2%)	32 (33.0%)	37 (33.9%)	
M2 or distal MCA	297 (33.9%)	22 (22.7%)	18 (16.5%)	
Tandem occlusion	49 (5.6%)	11 (11.3%)	16 (14.7%)	
LKW to arrival in hours (median, IQR)	4.2 [1.6 - 10.9]	5.3 [1.6 - 11.0]	4.1 [1.8 - 11.1]	0.91
IV thrombolysis (n, %)	92 (10.5%)	13 (13.4%)	12 (11.0%)	0.68
LKW to IV thrombolysis in hours (median [IQR])	2.5 [1.6 - 3.4]	1.4 [1.2 - 2.7]	1.9 [1.6 - 2.9]	0.13
Endovascular treatment (n, %)	126 (14.4%)	4 (4.1%)	19 (17.4%)	0.01
LKW to groin puncture in hours (median [IQR])	5.5 [3.5 - 11.9]	7.5 [5.1 - 12.3]	8.4 [5.8 - 13.5]	0.20
Vascular risk factors (n, %)				
Hypertension	518 (59.1%)	66 (68.0%)	81 (74.3%)	<0.01
Diabetes	245 (28.0%)	34 (35.1%)	36 (33.0%)	0.22
Dyslipidemia	218 (24.9%)	29 (29.9%)	29 (26.6%)	0.54
Smoking	333 (38.0%)	40 (41.2%)	38 (34.9%)	0.64
Atrial fibrillation	246 (28.1%)	18 (18.6%)	40 (36.7%)	0.02
Baseline imaging ratings				
ASPECTS (median [IQR])	10 [9 - 10]	9 [8 - 10]	10 [8 - 10]	0.17
Advanced WMH (n, %)	235 (27.3%)	26 (27.1%)	41 (39.0%)	0.04
Old infarction, all (n, %)	296 (34.4%)	33 (34.4%)	44 (41.9%)	0.31
CMB, all (n, %)	97 (11.3%)	10 (10.4%)	10 (9.5%)	0.85
Collateral grade (n, %)				0.75

poor (0, 1)	55 (6.7%)	8 (8.4%)	8 (7.9%)	
intermediate (2, 3)	307 (37.1%)	31 (32.6%)	41 (40.6%)	
good (4, 5)	465 (56.2%)	56 (58.9%)	52 (51.5%)	
Stroke outcomes				
Any hemorrhages on the follow-up image (n, %)	131 (15.0%)	20 (20.6%)	21 (19.3%)	0.21
Significant hemorrhage	32 (3.7%)	9 (9.3%)	10 (9.2%)	<0.01
Duration of hospital stay in days (median [IQR])	6.4 [4.7 - 9.4]	9.6 [6.3 - 15.2]	9.6 [6.2 - 16.2]	<).01
mRS 0 - 1 at 3 months (n, %)	521 (60.0%)	37 (38.9%)	18 (16.8%)	<0.01
mRS 0 - 2 at 3 months (n, %)	675 (77.8%)	58 (61.1%)	33 (30.8%)	<0.01
Death at 3 months (n, %)	21 (2.4%)	2 (2.1%)	13 (12.1%)	<0.01

* Early neurological deterioration (END) not related to EVT consisted of neurological deterioration before EVT initiation or in medically treated patients. Categorical variables are summarized as frequencies (percentages) and continous variables as mean ± SD or median [IQR_{25}- IQR_{75}]
Significant hemorrhage comprises PH2 hemorrhagic transformation and HBC class II.

Among 207 patients who developed END, EVT was performed in 23 (11.1%) cases. The NIHSS score on the END was a median of 9 [7 - 13] in EVT cases and a median of 7 [4 - 10] in medically managed patients, without statistically significant difference (*P*-value for difference, 0.27).

END was detected after a median of 24.5 [13.5 - 41.9] hours after the time last known well (LKW). END developed after a median of 6.7 [4.0 - 12.2] hours after the time LKW in the EVT group and after a median of 26.2 [16 - 44] hours after the time LKW in the medically managed group (P-value for difference, <0.01).

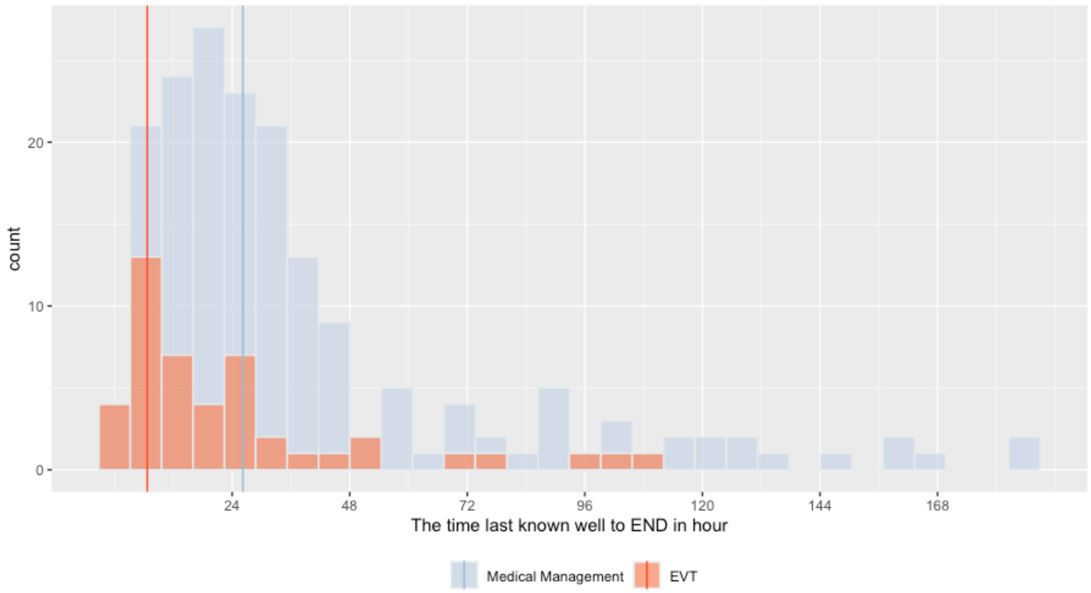

Therefore, among the cases with END, EVT was initiated after a median of 8.2 [5.8 - 13.5] hours after the time LKW and a median of 4.8 [2.6 - 10.5] hours after the arrival.

Excellent functional recovery of mRS 0 - 1 at three months after stroke was found in 55 (27.1%) cases with END and 521 (60.0%) cases without END. The proportions of mRS 0 - 1 after END were 45.5% (10 out of 22 available cases) in EVT cases and 24.9% (45 out of 181 available cases) in the medical management group. mRS at three months was not recorded in 4 cases who had END. There was no significant difference in the proportion of excellent functional recovery by EVT after the END (45.5%; 10 out of 22 cases) and EVT without the preceding END (49.6%; 62 out of 125 cases).

Supplemental Data II. List of the Clinical Research Collaboration for Stroke in Korea investigators

Seoul National University Bundang Hospital, Seongnam-si (Coordinating site)	Hee-Joon Bae, Moon-Ku Han, Jihoon Kang, Beom Joon Kim, Jun Yup Kim, Keon-Joo Lee
Eulji General Hospital, Seoul	Jong-Moo Park, Kyusik Kang, Inyoung Chung
Eulji University Hospital, Daejeon	Soo Joo Lee, Jae Guk Kim
Dong-A University Hospital, Busan	Jae-Kwan Cha, Dae-Hyun Kim, Jin-Heon Jeong
Seoul Medical Center, Seoul	Tai Hwan Park, Sang-Soon Park
Soonchunhyang University Hospital, Seoul	Kyung Bok Lee
Yeungnam University Medical Center, Daegu	Jun Lee, Doo Hyuk Kwon
Inje University Ilsan Paik Hospital, Goyang-si	Keun-Sik Hong, Yong-Jin Cho, Hong-Kyun Park
Hallym University Sacred Heart Hospital, Anyang-si	Byung-Chul Lee, Kyung-Ho Yu, Mi-Sun Oh, Minwoo Lee
Dongguk University Ilsan Hospital, Goyang	Dong-Eog Kim, Wi-Sun Ryu
Chonnam National University Hospital, Gwangju	Joon-Tae Kim, Man-Seok Park, Kang-Ho Choi, Ki-Hyun Cho
Jeju National University Hospital, Jeju	Jay Chol Choi, Joong-Goo Kim, Chul Hoo Kang
Ulsan University Hospital, Ulsan	Jee-Hyun Kwon, Wook-Joo Kim
Chungbuk National University Hospital, Cheongju-si	Dong-Ick Shin, Kyu Sun Yum, Baik-Kyun Kim
Keimyung University Dongsan Medical Center, Daegu	Sung-Il Sohn, Jeong-Ho Hong, Hyungjong Park
Hallym University Chuncheon Sacred Heart Hospital, Chuncheon-si	Chulho Kim, Sang-Hwa Lee
Chung-Ang University Hospital, Seoul	Kwang Yeol Park, Hae-Bong Jeong
Asan Medical Center, Seoul	Ji Sung Lee
Department of Biostatistics, Korea University, Seoul	Juneyoung Lee

Supplemental Data III. STROBE Statement

STROBE Statement—checklist of items that should be included in reports of observational studies

	Item No	Recommendation	Page No
**Title and abstract**	1	(*a*) Indicate the study's design with a commonly used term in the title or the abstract	4
		(*b*) Provide in the abstract an informative and balanced summary of what was done and what was found	4
**Introduction**			
Background/rationale	2	Explain the scientific background and rationale for the investigation being reported	6
Objectives	3	State specific objectives, including any prespecified hypotheses	6
**Methods**			
Study design	4	Present key elements of study design early in the paper	7
Setting	5	Describe the setting, locations, and relevant dates, including periods of recruitment, exposure, follow-up, and data collection	7
Participants	6	(*a*) *Cohort study*—Give the eligibility criteria, and the sources and methods of selection of participants. Describe methods of follow-up<br>*Case-control study*—Give the eligibility criteria, and the sources and methods of case ascertainment and control selection. Give the rationale for the choice of cases and controls<br>*Cross-sectional study*—Give the eligibility criteria, and the sources and methods of selection of participants	7
		(*b*) *Cohort study*—For matched studies, give matching criteria and number of exposed and unexposed<br>*Case-control study*—For matched studies, give matching criteria and the number of controls per case	Supplemental Method 1
Variables	7	Clearly define all outcomes, exposures, predictors, potential confounders, and effect modifiers. Give diagnostic criteria, if applicable	7
Data sources/ measurement	8*	For each variable of interest, give sources of data and details of methods of assessment (measurement). Describe comparability of assessment methods if there is more than one group	*7*
Bias	9	Describe any efforts to address potential sources of bias	8

Study size	10	Explain how the study size was arrived at	7
Quantitative variables	11	Explain how quantitative variables were handled in the analyses. If applicable, describe which groupings were chosen and why	8-9
Statistical methods	12	(*a*) Describe all statistical methods, including those used to control for confounding	8
		(*b*) Describe any methods used to examine subgroups and interactions	8
		(*c*) Explain how missing data were addressed	8
		(*d*) *Cohort study*—If applicable, explain how loss to follow-up was addressed<br>*Case-control study*—If applicable, explain how matching of cases and controls was addressed<br>*Cross-sectional study*—If applicable, describe analytical methods taking account of sampling strategy	8
		(*e*) Describe any sensitivity analyses	8-9

Continued on next page

**Results**			
Participants	13*	(a) Report numbers of individuals at each stage of study—eg numbers potentially eligible, examined for eligibility, confirmed eligible, included in the study, completing follow-up, and analysed	7
		(b) Give reasons for non-participation at each stage	7
		(c) Consider use of a flow diagram	Supplemental Method 1
Descriptive data	14*	(a) Give characteristics of study participants (eg demographic, clinical, social) and information on exposures and potential confounders	9
		(b) Indicate number of participants with missing data for each variable of interest	7
		(c) *Cohort study*—Summarise follow-up time (eg, average and total amount)	N/A
Outcome data	15*	*Cohort study*—Report numbers of outcome events or summary measures over time	
		Case-control study—Report numbers in each exposure category, or summary measures of exposure	
		Cross-sectional study—Report numbers of outcome events or summary measures	*9*
Main results	16	(*a*) Give unadjusted estimates and, if applicable, confounder-adjusted estimates and their precision (eg, 95% confidence interval). Make clear which confounders were adjusted for and why they were included	9-10
		(*b*) Report category boundaries when continuous variables were categorized	9-10
		(*c*) If relevant, consider translating estimates of relative risk into absolute risk for a meaningful time period	9-10
Other analyses	17	Report other analyses done—eg analyses of subgroups and interactions, and sensitivity analyses	10
**Discussion**			
Key results	18	Summarise key results with reference to study objectives	10-11
Limitations	19	Discuss limitations of the study, taking into account sources of potential bias or imprecision. Discuss both direction and magnitude of any potential bias	12
Interpretation	20	Give a cautious overall interpretation of results considering objectives, limitations, multiplicity of analyses, results from similar studies, and other relevant evidence	12
Generalisability	21	Discuss the generalisability (external validity) of the study results	10-12
**Other information**			
Funding	22	Give the source of funding and the role of the funders for the present study and, if applicable, for the original study on which the present article is based	13

*Give information separately for cases and controls in case-control studies and, if applicable, for exposed and unexposed groups in cohort and cross-sectional studies.

Note: An Explanation and Elaboration article discusses each checklist item and gives methodological background and published examples of transparent reporting. The STROBE checklist is best used in conjunction with this article (freely available on the Web sites of PLoS Medicine at http://www.plosmedicine.org/, Annals of Internal Medicine at http://www.annals.org/, and Epidemiology at http://www.epidem.com/). Information on the STROBE Initiative is available at www.strobe-statement.org.
